# Bioactive Potential of Olive Mill Waste Obtained from Cultivars Grown in the Island of Malta

**DOI:** 10.3390/foods13081152

**Published:** 2024-04-10

**Authors:** Frederick Lia, Karen Attard

**Affiliations:** Institute of Applied Science, Malta College of Arts, Science and Technology, PLA 9032 Paola, Malta; karen.attard.b42517@mcast.edu.mt

**Keywords:** cell lines, polyphenols, allelopathy, toxicity, olive pomace

## Abstract

This study explores the bioactive potential of olive mill waste derived from cultivars grown in the Maltese Islands through various analytical approaches. Cell culture, cell staining, allelopathic assays, shrimp brine lethality assays, and HPLC analysis were conducted to assess the efficacy and bioactivity of the extracts using different treatments, including methanolic extraction, acid, and alkaline hydrolysis. Notably, the results from cell lines revealed that NB4r2 cells exhibited high susceptibility to the tested extracts, with the lowest IC_50_ recorded after 72 h of exposure. Notably, the ‘Bajda’ cultivar displayed the most effectiveness, particularly with acid hydrolysis. In allelopathic assays, higher concentrations of ‘Malti’, ‘Bidni’, and ‘Bajda’ extracts significantly inhibited lettuce seed germination. Similarly, in the brine shrimp lethality assay, higher concentrations led to increased mortality rates of *Artemia salina*, though rates decreased at lower concentrations. The identification of phenolic compounds found in olive mill waste was conducted using high-performance liquid chromatography (HPLC) with the use of internal standards. The identification revealed a variety of compounds, with 3-hydroxytyrosol and oleacein being present in high abundance in nearly all hydrolyzed and methanolic extracts, whereas gallic acid was found to be the least abundant. These findings highlight the rich bioactive potential of olive mill waste and provide insights into its applications in pharmaceuticals, nutraceuticals, and agriculture, emphasizing the importance of further research to fully exploit these valuable resources.

## 1. Introduction

In recent years, the growing interest in sustainable agricultural methods has spurred efforts to manage waste effectively and extract valuable bioactive compounds from by-products [1]. Olive mill waste, abundant in bioactive compounds, poses environmental concerns due to its potential to release harmful substances into soil and water sources [2]. This waste is rich in phenolic compounds, with only a small fraction (2%) being transferred to olive oil during production, leaving the majority in the waste [3]. Phenolic compounds found in olive pomace encompass phenolic alcohols, phenolic acids, lignans, secoiridoid derivatives, and flavonoids [4], which are known for their bioactivity and health benefits, including antioxidant, anti-inflammatory, and antimicrobial properties [5]. Thus, utilizing olive mill waste not only offers an alternative to environmental pollution but also presents an opportunity to harness its bioactive potential.

Over the past few years, extensive research has been conducted on the health benefits and anticancer effects of plant-derived polyphenols [6]. Studies demonstrated that combinations of polyphenols can exhibit synergistic effects, enhancing their biological activities beyond those of individual compounds [7,8,9]. Furthermore, these combinations can improve the bioavailability and solubility of polyphenols, enhancing their efficacy in various therapeutic applications [10]. Several studies showed that natural polyphenols, when combined with chemotherapeutics, can reduce side effects, increase anticancer efficacy, and overcome resistance in cancer cells [11,12]. Phenolic compounds extracted from olive oil waste were shown to exert possible chemoprotective and anticancer activities in different types of cancer cells [13,14]: prostate [15], breast cancer [16], colon [17], promyelocytic leukemia [18], melanoma [19], and other cancer cells.

The primary source of toxicity in olive mill waste arises from its elevated organic content, posing a threat of environmental contamination if not effectively managed. A key method utilized for the initial assessment of toxicity in various substances, including plant extracts [20,21], heavy metals [22], fungal toxins [23], pesticides [24], and cyanobacteria toxins [25], is the brine shrimp lethality assay (BSLA). This assay serves to detect harmful substances present in plant extracts, capable of inducing mortality in laboratory-reared nauplii. Indeed, numerous studies demonstrated the lethal impact of olive mill waste on brine shrimp larvae [26,27]. *Artemia salina*, commonly used in the BSLA, is a zooplanktonic crustacean abundant in lakes and oceans, serving as a staple food for numerous aquatic organisms. Due to its frequent interaction with aquatic environments, *Artemia salina* faces heightened exposure to contaminants [28]. Notably, nauplii exhibit greater sensitivity to toxic agents compared to adult *Artemia salina* [29]. *Artemia salina* serves as a widely adopted model organism for toxicological assessments due to its ease of cultivation, rapid life cycle, commercial availability of cysts, prolific offspring production, affordability, year-round accessibility, and safety considerations [28,30,31].

Olive mill waste poses a significant challenge in Mediterranean regions due to its elevated lipid, phenol, and organic acid concentrations, rendering it phytotoxic and potentially detrimental to soil fertility [32]. Therefore, careful consideration is necessary when applying olive mill waste to crops, as it may entail negative effects and limitations that could impact nearby plants. A crucial process within plant communities is allelopathy, wherein one plant, including microorganisms, exerts both direct and indirect effects on another by releasing chemical compounds into the environment, known as allelochemicals [33]. These compounds, typically emitted through root exudation, leaching, residue decomposition, and volatilization, can influence various stages of plant growth and development, such as seed germination, flowering, fruiting, seedling growth, succession, vegetation formation, and species regeneration [34,35,36,37,38]. The presence of these allelochemicals can disrupt essential physiological processes like respiration, photosynthesis, water regulation, and hormonal balance, thereby impacting the germination and growth of neighboring plants [39].

The overall objective of this study is to determine the bioactive potential of olive mill waste against human cancer cell lines and identify its effect on germination and plant growth together with the identification of potential toxicity towards brine shrimp lethality assay using *Artemia salina*. Additionally, the study aims to identify phenolic compounds present in the waste using HPLC.

## 2. Materials and Methods

### 2.1. Collection and Extraction of Raw Materials 

Samples of olive mill waste were collected and treated following the methodology outlined in a prior publication referenced in Section 2.1 [40]. Three distinct Maltese cultivars, namely ‘Bidni’, ‘Bajda’, and ‘Malti’, were collected, filtered, freeze-dried, and ground into powder. Subsequently, the samples underwent extraction and hydrolysis procedures. Three separate extraction methods were employed: initial extraction with methanol (AR Biochem Chemopharma, Cosne-Cours-sur-Loire, France), followed by acid hydrolysis using 6 M HCl (VWR), and alkaline hydrolysis using 10 M NaOH (Biochem Chemopharma, Cosne-Cours-sur-Loire, France). The hydrolyzed samples were pH-adjusted to 3 and subjected to ethyl acetate extraction (Biochem Chemopharma, Cosne-Cours-sur-Loire, France) three times the sample volume. Both ethyl acetate and methanolic fractions were concentrated through evaporation under reduced pressure (IKA^®^ RV10) and further concentrated under a gentle nitrogen stream (Organ-mations N-EVAP^®^ 112). The total phenolic content and corresponding antioxidant activity were detailed in the preceding article, Section 2.3, Section 2.4, Section 2.5, Section 2.6, Section 2.7, Section 2.8, Section 2.9, Section 2.10 and Section 2.11 [40]. 

### 2.2. Cells

Three non-adherent cell lines, comprising human promyelocytic leukemia (HL-60) CCL-240, promyeloblast macrophage cell line (KG-1a) CCL-246.1, and ATRA-resistant NB4r2 cells, were cultured in RPMI medium. All cell lines were procured from ATCC. These non-adherent cell lines were supplemented with fetal bovine serum (Gibco™) and Penicillin–Streptomycin (10,000 U/mL) (Gibco™) penicillin. Subsequently, these cells were incubated in a CO_2_ incubator (Memmert Gmbh, Pune, India) at 37 °C with 5% CO_2_. The culture medium was refreshed every 3–4 days. Cell counting was conducted using a hemocytometer with Trypan blue (Sigma-Aldrich, Steinheim, Germany) as an indicator, and the resulting single-cell suspension was diluted to 8000 cells per well in 180 mL of medium.

### 2.3. MTT Assay

The cytotoxic potential of extracts derived from olive mill waste was assessed by using the MTT assay [41]. A volume of 180 mL of cells was dispensed into each well of a 96-well plate. These plates were then incubated at 37 °C for 24 h to allow the cells to acclimatize. Subsequently, 30 μL aliquots of methanolic and hydrolyzed extracts from the ‘Bidni’, ‘Bajda’, and ‘Malti’ cultivars were added. Moreover, 4 h before the termination of each exposure time (24, 48, 72 h), 35 μL aliquot of MTT was added to each well. These were left to incubate for 4 h at 37 °C. Upon completion of the incubation period, the cells were examined for the development of a dark blue formazan coloration. The plates were then subjected to centrifugation at 4500 rpm for 5 min, and the supernatant was emptied with one quick stroke. This was followed by the addition of pipetting 25 μL of Sorenson’s Glycine Buffer and 200 μL of DMSO (Biochem Chemopharma) to each well. The absorbance was read at 630 nm.

### 2.4. Cell Staining 

Three different staining procedures were conducted in order to visualize the cells. Initially, for Giemsa staining (Sigma), the slides were left to air dry and then immersed in Giemsa staining for one minute, followed by immersion in water. For hematoxylin and eosin staining (Carl Roth), air-dried slides were rehydrated in decreasing ethanol concentrations (100%, 80%, and 70%) (Sigma) for 5 min each. After a tap water rinse, slides were immersed in hematoxylin for 15 min, rinsed for 2 min, and subjected to acid de-staining, repeated five times. Subsequently, slides were immersed in bluing agent, rinsed, and placed in methanol. The counter-staining was carried out by immersing the slides in a 1 g/L eosin solution for 2 min, followed by three washes in 95% ethanol. The slides were air-dried, washed with xylene (Sigma), and air-dried again [42]. As for Quinacrine Dihydrochloride Fluorescent Staining, air-dried slides were rehydrated in McIlvain’s buffer for 5 min, immersed in a 0.5% quinacrine dihydrochloride (Sigma) solution for 20 min, rinsed, and mounted with McIlvaine’s buffer (Fisher Scientific, Waltham, MA, USA) for observation under a fluorescent microscope (Olympus) using a blue filter. After air-drying, all slides were mounted in Canada Balsam (BDH) [43].

### 2.5. Preparation of Cells for Protein Assays

A total of 3 mL of cell suspension was dispensed into each well of a 6-well plate. After 24 h, various concentrations of ‘Bidni’, ‘Bajda’, and ‘Malti’ extracts were added to the cells. Following an additional 24 h incubation period at 37 °C, the cell-extract mixtures from each well were scraped and transferred to centrifuge tubes, dividing them into three equal portions of 1 mL each. The centrifuge tubes underwent centrifugation, after which the media were carefully removed. The cell layer was subsequently washed twice with sterile PBS. This procedure was carried out for Section 2.6, Section 2.7, and Section 2.8. 

### 2.6. Apoptosis DNA 

DNA laddering assay was conducted by using the Apoptosis DNA Ladder Assay kit sourced from Abcam (ab66090). Following the PBS washing in Section 2.5, 30 μL of TE lysis buffer was added to lyse cells. Subsequently, 5 μL of Enzyme A and 5 μL of Enzyme B were added. The solutions underwent centrifugation and were stored in a −80 °C freezer. After 24 h, the supernatant was transferred to a new centrifuge tube, followed by the addition of 5 μL of ammonium acetate solution and 100 μL of IPA. The tubes were centrifuged, and the supernatant was discarded, leaving the DNA pellet visible at the bottom. The DNA pellet was washed with 200 μL of 70% ethanol and centrifuged again. The ethanol was decanted, and the centrifuge tube was dried in a heating block. After 24 h, the DNA pellet was dissolved in 30 μL of DNA suspension buffer. 

#### Gel Electrophoresis

A total of 1.2 g of agarose (Biochem Chemopharma) was dissolved in 100 mL of TE × 1 buffer, supplemented with 100 μg of propidium iodide. After solidification of the gel, a voltage of 5 V/cm was applied. Subsequently, the extract samples from Section 2.6 were loaded into the wells of the gel. Once the process was completed, the bands were visualized through transillumination using UV light (UVP).

### 2.7. Protein Content

Protein content was determined using a bovine serum albumin standard at a concentration of 2 mg/mL. A calibration curve ranging from 2 to 0.01 mg/mL was conducted by pipetting 10 μL of each concentration into a 96-well plate, followed by the addition of 250 μL of Bradford reagent at a dilution factor of 10. The plate was then read at a wavelength of 595 nm. The calibration curve equation was y = 0.9903x + 0.3853, with an R^2^ value of 0.9934, as shown in Supplementary Material Figure S1. The protein content of the samples was initially assessed by thawing the cells and subjecting them to centrifugation to obtain the supernatant. Subsequently, 5 μL of the supernatant was aliquoted into a 96-well plate, supplemented with 5 μL of TEx1 buffer to achieve a dilution factor of 2. Following this, 250 μL of Bradford reagent diluted tenfold was pipetted, and the absorbance of the plate was measured at 595 nm.

#### Caspase-3 Assay

Caspase-3 protein quantification was conducted using the Caspase-3 Assay Kit (Colorimetric) sourced from Abcam (ab39401). Following PBS washing as outlined in Section 2.5, 50 μL of chilled lysis buffer was added, and cells were allowed to incubate on ice for 10 min. Subsequent to centrifugation, the supernatant was carefully transferred to a fresh tube. The protein content obtained from Section 2.7 was quantified and adjusted to a range of 50–200 μg. Moreover, 10 μL of the extract was pipetted into a 96-well plate, followed by the addition of 50 μL of 2× buffer containing 10 mM DTT and 5 μL of DEVD-p-NDA. The plate underwent vigorous shaking and was then incubated for 1–2 h at 37 °C. After this incubation period, the plate was read at 400–405 nm using a microplate reader.

### 2.8. BCL-2 and BAX Protein Assays

BCL-2 quantification was performed using Human BCL-2 SimpleStep Elisa^®^ Kit from Abcam (ab272102). A calibration curve ranging from 12,500 to 195.31 pg/mL was conducted. The calibration curve equation, y = 0.0001x + 0.1644, with an R^2^ value of 0.9987, was established and can be found in the Supplementary Material Figure S2. For BAX quantification, Human BAX SimpleStep ELISA kit provided by Abcam (ab199080) was used. A calibration curve ranging from 800 to 12.5 pg/mL was conducted. The calibration curve equation, 0.9976x + 0.1477, with an R^2^ value of 0.9976, was determined and can be seen in the Supplementary Material Figure S3. Subsequently, for both BAX and BCL-2 assay, 40 μL of sample and standard were pipetted into a 96-well plate, followed by the addition of 50 μL pre-made antibody cocktail. This cocktail consisted of 300 μL of 10× capture antibody, 300 μL 10× detector antibody, and 2.4 mL of antibody diluent 5BI. The plate was sealed and incubated for 1 h at room temperature on a plate shaker set to 400 rpm. Following incubation, the plate underwent three washes with 350 μL of 1× wash buffer solution, ensuring complete drying after the final wash. Subsequently, 100 μL of TMB development solution was added and incubated for 10 min in the dark on a plate shaker at 400 rpm. After 10 min, 100 μL of stop solution was added, and the plate was left to incubate for 24 h. After 24 h, the plate was read at 450 nm.

### 2.9. Brine Shrimp Lethality Assay 

The brine shrimp lethality assay was conducted following the methodology outlined by Bastos et al. [44]. Brine shrimp eggs were hatched for 24 h in a separating funnel containing 300 mL of 37% saline water, facilitated by the presence of spirulina and yeast. The separating funnel was adequately aerated using an air pump, and a continuous bright light source was maintained, resulting in nauplii hatching within 24 h. The stock solution was dissolved in 50:50 saline water and ethanol whilst the dilutions were dissolved in 37% saline water. After 24 h of incubation, approximately 100 μL of solution was pipetted into 24-well plates, each containing 10 nauplii. Subsequently, an additional 800 μL of 37% saline water was added to all wells. A total of 180 μL of previously prepared diluted extracts were pipetted into the 24-well plate. The plates were then left uncovered for 24 h at 25 °C under constant illumination. After this incubation period, the *Artemia salina* were counted under a microscope.

The lethal concentration (LC_50_) for brine shrimp with 95% confidence level was determined by Probit analysis. Percentage mortalities were corrected for natural mortality observed in the negative controls using Abbott’s Formula (1).

(1) $$P=\frac{P_{i}-C}{1-C}$$

where P_i_ is the observed mortality rate, and C is the natural mortality.

### 2.10. Allelopathic Assay 

The seed germination method employed closely follows the methodology outlined by Zhao et al. [45]. Lettuce seeds were purchased and stored via refrigeration at 5 °C to 10 °C. Prior to experimentation, the lettuce seeds underwent careful selection and immersion in distilled water for 24 h, followed by sterilization using a 0.3% KMnO_4_ (Biochem Chemopharma) solution for 15 min. Post-sterilization, the seeds were washed thrice with distilled water and air-dried. The 15 cm diameter petri dishes and the 0.2% agarose were autoclaved at 105 °C for 20 min before use. Subsequently, 2 mL of each previously prepared extract concentration, dissolved in 0.7% agarose, was pipetted directly into the agar. Upon agar solidification, approximately 20 seeds were uniformly distributed in each petri dish. All petri dishes were then placed in an illuminated environmental incubator set at a constant temperature of 25 °C and humidity level of 90%. Germination of lettuce seeds was monitored and recorded for a duration of 11 days.

#### Calculations

Germination rate (GR), germination energy (GE), germination index (GI), vigor index (VI), and mean germination time (MGT) were calculated by using Equation (2)–(6) [46,47].

(2) $$GR\left(\%\right)=\frac{Ni}{N}\times 100$$

(3) $$GE\left(\%\right)=\frac{Nt}{N}\times 100$$

(4) GI=∑(d/n)

(5) VI=GR×(LR+LP)

(6) $$MGT=\frac{\sum \;(d*n)}{\sum n}$$

where Ni represents the count of germinated seeds on the 11th day, N is the total number of seeds in the petri dish, Nt indicates the number of germinated seeds at the peak daily germination, d signifies the number of seeds emerging on a specific day, n refers to the time elapsed since setting the seeds for germination, LR stands for the average length of the radicle on the 11th day, and LP represents the average length of the plumule on the 11th day.

The allelopathy index (RI) was calculated by using Equation (7) [48].

(7) $$RI\left(\%\right)=\frac{\left(T-C\right)}{C}\times 100$$

where T is the treatment value, and C is the control value. 

### 2.11. HPLC Profiling 

All standards and samples were initially prepared and filtered through PVDF 0.45 µm syringe filters. Alongside the standards (purity between 85.18% and 99.99%), 250 µL of 100 μg/mL syringic acid (Sigma) was added as an internal standard to 500 µL of the standard solution. A total of 25 different standards were utilized and purchased from Sigma, Alfa Aesar, PhytoLab, Honeywell Fluka™, and Carl ROTH. 

The HPLC system used (Shimadzu LC-20 AB, Kyoto, Japan) was equipped with a binary pump, autosampler (SIL-20AC), and UV/vis detector (SPD-20AV), monitoring at 280 nm and 320 nm. Analysis was conducted with a 20 μL injection volume on an ACE^®^ C18 analytical column (250 × 4.6 mm i.d.) with a 5 μm particle size (Aberdeen, Scotland). Mobile phases (A) consisted of 0.2% trifluoroacetic acid and (B) methanol/acetonitrile at a constant flow rate of 1 mL/min. The gradient elution followed a specified program: starting with 95% (A); 5% (B) for 0 to 30 min, transitioning to 70% (A); 30% (B) from 30 to 35 min, further to 50% (A); 50% (B) from 35 to 40 min, reaching 100% (B) from 40 to 50 min, and finally, returning to 95% (A); 5% (B) for the last 2 min as a post-equilibration step. Column temperature was maintained at 35 °C with a limit of 40 °C, controlled by a Shimadzu CTO-10AC thermostatically controlled column compartment, while the sample chamber was kept at 4 °C to prevent degradation. Detector acquisition time was 110 min, with an LC stop time of 117 min. 

The compound quantification, following phenolic identification through RRT, utilized a 6-point calibration curve ranging from 0.1 to 100 µg/mL. System suitability was assessed with 6 injections of syringic acid at 50 µg/mL, requiring an acceptance criterion of RSD < 5%. Intermediate precision was evaluated by injecting syringic acid at 50 µg/mL after every 10 samples, measured in duplicates. The quantification involved determining the linearity, recovery, LOQ, and LOD of individual compounds using externally prepared calibration curves. Compounds were identified through peak identification, standards, and relative retention time (RRT), as detailed in Supplementary Material Table S1.

### 2.12. Data Analysis

Univariate data analysis was conducted using SPSS Inc. Version 16.0 (Chicago, IL, USA). The data derived from various assays served as independent variables, while the cultivar type and sample treatment were treated as categorical variables. Normality testing was performed using both the Kolmogorov–Smirnov test and the Shapiro–Wilk test (SPSS Inc.). In instances where the null hypothesis was rejected (*p*-value < 0.05), non-parametric tests were employed. Kruskal–Wallis one-way analysis of variance by ranks test was utilized for the analysis of variance, followed by the Mann–Whitney U test for pairwise comparisons. For chemometric analysis, a target-based analytical approach was adopted, utilizing the concentrations of specified compounds identified via HPLC. Data underwent direct importation into The Unscrambler X 10.3 (CAMO Software, Oslo, Norway) for normalization and standardization procedures. Principal Component Analysis (PCA), two-way cluster analysis using Ward’s method, and matrix correlation analysis using Spearman correlation coefficient were performed using JMP 10 (SAS) to extract insights, identify patterns, and discern relationships within the dataset.

## 3. Results

### 3.1. Non-Adherent Cell Lines–Add Values 

#### 3.1.1. Effect of Bioactivity on Cancer Cell Lines

**Figure 1:**
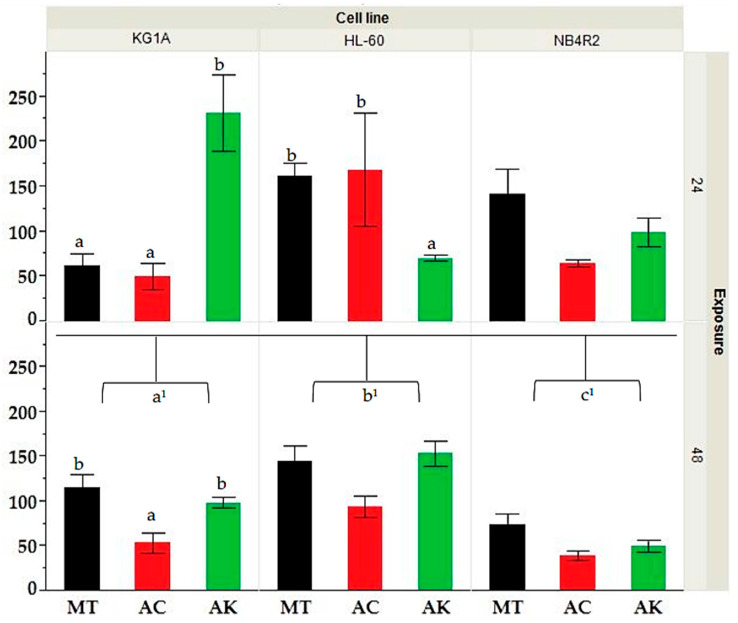
Comparative analysis of IC_50_ values for HL-60, KG-1a, and NB4r2 cell lines following 24 and 48 h of exposure for extracts derived from different treatments (MT = methanol, AC= acid, AK = alkaline). Different letters (a, b and c) indicate significant difference (*p*-value ≤ 0.05) by Kruskal–Wallis ANOVA followed by Man Whitney u test for differences between treatment whilst a^1^, b^1^, c^1^ indicate the significance between the IC_50_’s at 48 h of exposure between different cell lines.

The MTT assay was employed to assess the bioactivity of the various extracts against myeloid human cancer cell lines over three exposure times of 24, 48, and 72 h. The results presented in Supplementary Material Figure S4 showed that all extracts had a dose and time-dependent increase in bioactivity. HL-60 cells exhibited an average IC_50_ of 136.1 ± 42.1 at 24 h, which decreased to 127.4 ± 39.5 at 48 h and further to 102.4 ± 39.3 at 72 h. Similarly, KG-1a cells demonstrated a decline in IC_50_ over time, with averages of 113.2 ± 35.6, 90.4 ± 16.7, and 54.3 ± 18.7 at 24, 48, and 72 h, respectively. Notably, NB4r2 cells showed a significant decrease in IC_50_ from 105.4 ± 31.3 at 24 h to 59.0 ± 16.7 at 48 h and 40.3 ± 19.9 at 72 h, indicating their heightened susceptibility to the tested extracts compared to HL-60 and KG-1a cells. The application of Kruskal–Wallis ANOVA, followed by a Mann–Whitney U test shown in Figure 1, showed that the IC_50_ after 24 h exposure no significant difference was observed between cell lines. However, after 48 h of exposure, NB4r2 cell lines had a significantly lower IC_50_ compared to both HL-60 (*p*-value ≤ 0.01) and KG-1a (*p*-value = 0.01). Furthermore, KG-1a exhibited a significantly lower IC_50_ compared to HL-60 cell line (*p*-value = 0.022). All IC_50_ values were expressed in μg/mL. 

#### 3.1.2. Effect of Cultivars on Cell Lines to Different Extraction Methods 

The effect of cultivar on cell line response was investigated and can be seen in Supplementary Material Figure S5. For HL-60 cells, the ‘Malti’ cultivar exhibited the lowest IC_50_ values in alkaline hydrolysis (57.8 ± 42.0) and the highest in methanolic extraction (319.6 ± 53.4), while for ‘Bajda’, the lowest IC_50_ was observed in acid hydrolysis (23.4 ± 15.3) and the highest in alkaline hydrolysis (188.6 ± 60.6). ‘Bidni’ extract showed the lowest IC_50_ in methanolic extraction (37.2 ± 18.7) and the highest in acid hydrolysis (402.4 ± 76.7). Similarly, in KG-1a cells, the ‘Malti’ cultivar showed the lowest IC_50_ in acid hydrolysis (37.2 ± 17.3) and the highest in alkaline hydrolysis (280.3 ± 117.8), with similar trends observed for ‘Bajda’ (acid: 8.8 ± 6.6 and alkaline: 270.7 ± 31.1) and ‘Bidni’ (methanol: 40.8 ± 34.1 and alkaline: 109.6 ± 7.6) extracts. NB4r2 cells exhibited the lowest IC_50_ for the ‘Malti’ cultivar in acid hydrolysis (16.7 ± 10.1) and the highest in methanolic extraction (265.3 ± 73.7), while for ‘Bajda’, the lowest IC_50_ was found in acid hydrolysis (3.5 ± 2.5) and the highest in methanolic extraction (92.3 ± 45.4). ‘Bidni’ extract displayed the lowest IC_50_ in alkaline hydrolysis (13.9 ± 6.7) and the highest in methanolic extraction (100.3 ± 15.0). All data were expressed in μg/mL. From the results obtained, acid hydrolysis exerted the most significant effect on the cell lines, followed by alkaline and methanolic extraction, with statistical significance indicated by a *p*-value of less than 0.05. Specifically, the ‘Bajda’ extract exhibited the highest efficacy.

A Kruskal–Wallis ANOVA, followed by a Mann–Whitney U test analysis shown in Figure 1, showed that KG-1a cell lines at 24 h indicated a significant difference between the extracts of ‘Bidni’ and ‘Malti’ (*p*-value = 0.007), where the ‘Bidni’ extract exhibited the lowest IC_50_ median of 9.00, while ‘Malti’ showed the highest IC_50_ median of 20.33. Additionally, for the treatment methods on the KG-1a cell line at 24 h a significant difference between acid and alkaline-hydrolyzed extracts (*p*-value = 0.025), as well as between methanol and alkaline extracts (*p*-value = 0.027), was seen. Specifically, acid hydrolysis showed the lowest IC_50_ median of 10.67, whereas alkaline hydrolysis demonstrated the highest IC_50_ median of 20.56. The Kruskal–Wallis ANOVA showed no significance (*p*-value = 0.342) between the extracts obtained from different cultivars on the bioactivity observed after 48 h exposure for KG-1a cells. However, there was a significant difference between acid and methanolic extraction methods (*p*-value = 0.021), whilst no significant difference was observed between alkaline and methanolic extract (*p*-value = 0.722) and alkaline and acid hydrolyzed extracts (*p*-value = 0.413). Acid hydrolysis exhibited the lowest IC_50_ median of 8.78, while methanolic extraction displayed the highest IC_50_ median of 18.89. As for the HL-60 cell lines exposed for 24 h using extracts derived from different cultivars, no significant difference (*p*-value = 0.375) was observed. However, extracts obtained under alkaline hydrolysis exhibited a significantly lower IC_50_ compared to methanolic extraction (*p*-value = 0.005), which showed the highest IC_50_ median of 19.78. Lastly, for HL-60 cells at 48 h, and NB4r2 cells at both 24 and 48 h of exposure, there was no significant difference observed in both treatment and cultivar. All values in this section are expressed in μg/mL. 

### 3.2. Cell Staining 

**Figure 2:**
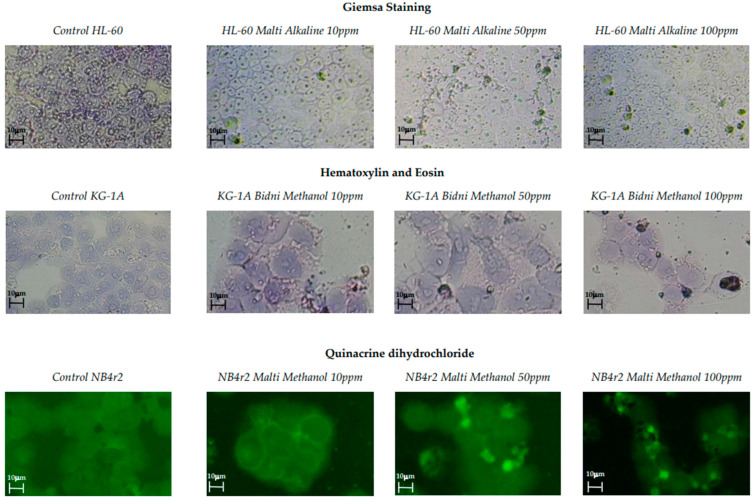
Giemsa staining, hematoxylin and eosin staining, and quinacrine dihydrochloride staining of olive mill waste extracts with concentrations of 100, 50, and 10 ppm with the addition of a control.

Three distinct cell staining methods were employed, including Giemsa staining, hematoxylin and eosin staining, and quinacrine dihydrochloride staining. These staining techniques were used to identify cellular morphological alterations and the visualization of apoptotic bodies. Figure 2 illustrates images of various staining techniques at different concentrations alongside a control for comparison. In all control samples, well-defined cellular structures were observed. It is evident that at 10 ppm, the cells exhibited a uniform shape with distinct nuclei and cell walls. However, at 100 ppm, the cellular shrinkage, apoptotic fragmentation, and chromatin condensation of the nucleus were observed. 

### 3.3. Apoptotic DNA Assay

**Figure 3:**
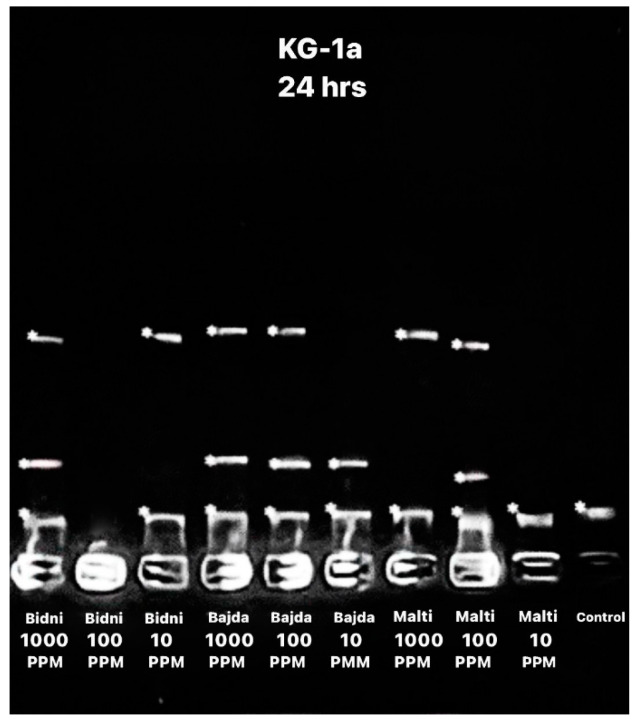
Apoptotic DNA assay showing the bend of DNA after 24 h of exposure using different extracts at different concentrations (1000, 100, and 10 ppm) with the addition of a control.

The apoptotic DNA assay performed after 24 h of exposure, as seen in Figure 3, revealed a higher number of bends at 1000 ppm when compared to 10 ppm. Specifically, the ‘Bidni’ and ‘Bajda’ cultivars exhibited three DNA bends at 1000 ppm, whereas the ‘Malti’ cultivar showed two bends. Conversely, at 10 ppm, two bends were observed for each ‘Bidni’ and ‘Bajda’, whereas ‘Malti’ had one bend.

### 3.4. Caspase-3 Activation 

As shown in the Supplementary Material Figure S6, the caspase-3 assay conducted on the KG-1a cell line indicated higher caspase-3 activity at 100 μg/mL. Specifically, the ‘Bidni’ cultivar exhibited the highest caspase-3 activity at both 100 and 10 μg/mL, while the lowest activity was observed in the ‘Malti’ extract at both concentrations. Notably, both concentrations displayed higher caspase-3 activation compared to the control. 

### 3.5. BAX and BCL-2

**Figure 4:**
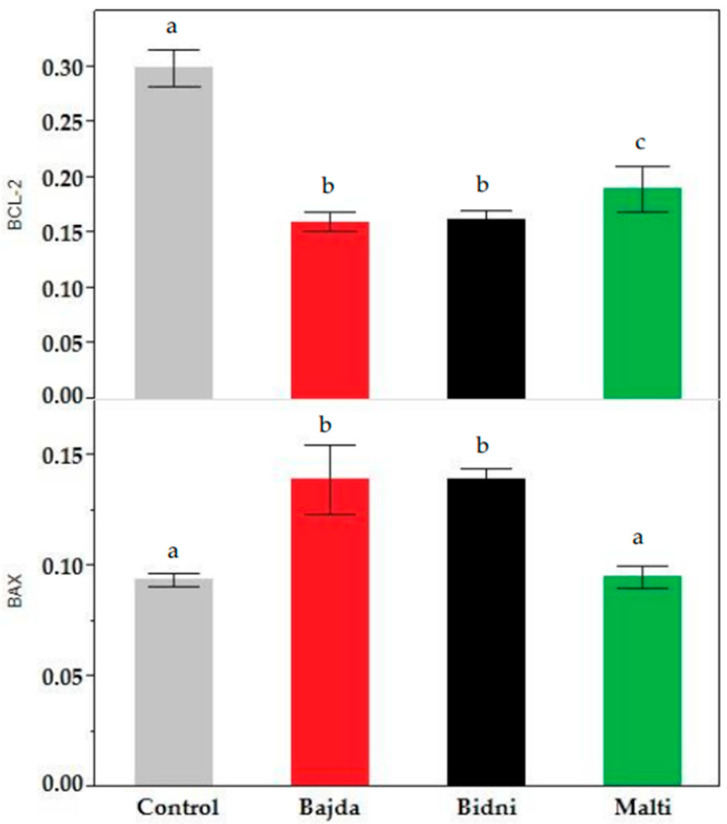
BAX (bottom) and BCL-2 (top) content in KG-1a cell lines after 48 h of exposure in ‘Bidni’, ‘Bajda’, and ‘Malti’ extracts compared to the control. For BAX content, a significant difference at 90% confidence level was observed between the control and ‘Bidni’ (*p*-value = 0.061), ‘Bajda’ (*p*-value = 0.080), and ‘Malti’ (*p*-value = 0.086) cultivars. Extracts from ‘Bidni’, ‘Bajda’, and ‘Malti’ cultivars in BCL-2 content showed a significant decrease compared to the control, with the former cultivars exhibiting *p*-values < 0.01, while for ‘Malti’, a *p*-value of 0.032 was obtained when compared to the control. Different letters (a, b and c) indicate significant difference (*p*-value ≤ 0.05) by Kruskal–Wallis ANOVA followed by Man Whitney u test.

The BCL-2 assay at 24 h of exposure presented in Supplementary Material Figure S7 revealed consistent findings between the 24 h and 48 h exposure time. Specifically, at 24 h, the BCL-2 content was notably higher than at 48 h; however, it remained lower than the control. For the 48 h exposure, it is evident that the ‘Bidni’ cultivar exhibited the highest BCL-2 content, while the ‘Bajda’ cultivar displayed the lowest. In fact, in comparison to the control, extracts derived from ‘Bidni’, ‘Bajda’, and ‘Malti’ cultivars showed a significant decrease from the control with *p*-values < 0.01. Similarly, the BAX assay results shown in Supplementary Figure S8 revealed higher BAX levels after 24 h of exposure compared to 48 h. At 48 h of exposure, the ‘Bajda’ extract exhibited the highest BAX concentration at 100 μg/mL, while the ‘Malti’ extract displayed the lowest concentration. Notably, the BAX content for both concentrations was higher than that of the control. A Kruskal–Wallis ANOVA, followed by the Mann–Whitney U test analysis shown in Figure 4, showed that extracts derived from ‘Bidni’, ‘Bajda’, and ‘Malti’ cultivars had no significant difference between the control at the 95% confidence interval; however, for ‘Bidni’ and ‘Bajda’, these observations were significant at the 90% confidence interval.

### 3.6. Allelopathic Assay 

The olive mill waste extracts had a significant impact on the germination rate, germination energy, and germination index of *Lactuca sativa* seeds. In general, higher concentrations of ‘Malti’, ‘Bidni’, and ‘Bajda’ extracts notably decreased lettuce seed germination, as detailed in Table 1. ‘Bidni’ alkaline extract exhibited the highest percentage germination rate (95.42 ± 0.81), while the lowest was observed in ‘Malti’ alkaline extract (26.67 ± 5.68). Regarding percentage germination energy, the ‘Bidni’ acid extract showed the highest percentage (80.00 ± 10.00), with the lowest found again in the ‘Malti’ alkaline extract (0.01 ± 0.00). Additionally, for the percentage germination index, the ‘Bidni’ alkaline extract had the highest percentage (95.42 ± 0.81), while the lowest was recorded in the ‘Malti’ alkaline extract (26.67 ± 5.68). 

The Kruskal–Wallis ANOVA results for the percentage germination energy (%GE) in the allopathic assay reveal no significant difference (*p*-value = 0.250) between cultivars. However, a significant difference (*p*-value = 0.002) was observed between alkaline and methanolic extracts, with alkaline extracts exhibiting the lowest %GE (7.00) and methanolic extraction yielding the highest %GE (20.00). Regarding the germination index (GI), the ‘Malti’ cultivar showed the lowest GI (8.76) followed by ‘Bidni’ (*p*-value = 0.015) and ‘Bajda’ (*p*-value = 0.045) extracts. Additionally, there was a significant difference between alkaline- and acid-hydrolyzed extracts (*p*-value = 0.027), with alkaline hydrolysis showing the lowest GI of 8.11 and acid-hydrolyzed extracts showing the highest GI of 17.89. Furthermore, the mean germination time (MGT) analysis indicated no significant difference between cultivars (*p*-value = 0.198). However, a significant difference (*p*-value = 0.011) was observed between alkaline and acid hydrolysis, with alkaline hydrolysis exhibiting the lowest MGT of approximately 10 days and acid hydrolysis exhibiting the highest MGT after approximately 21 days.

**Figure 5:**
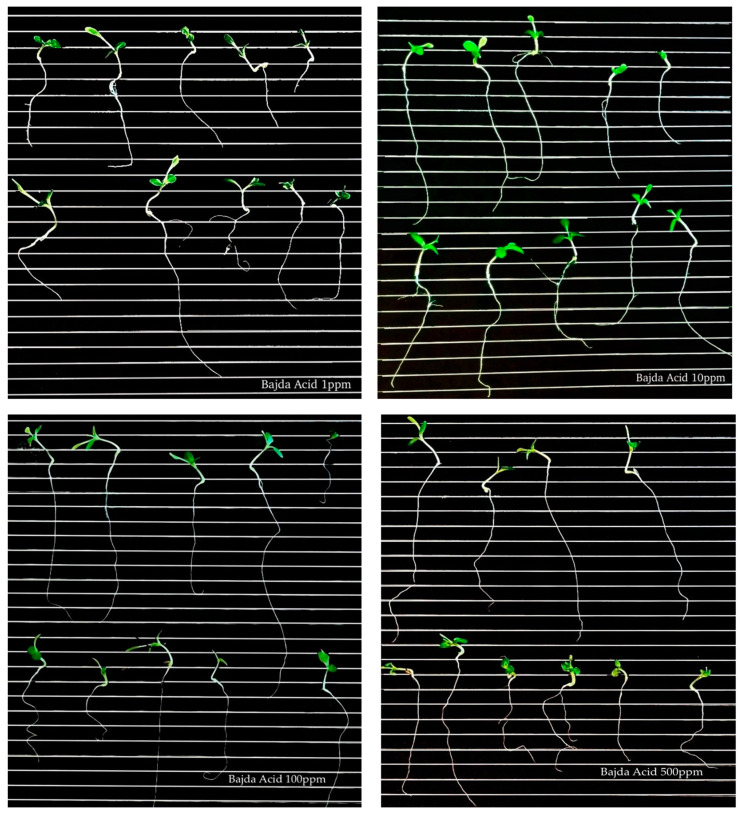
Germination of lettuce seeds treated with ‘Bajda’ acid extract at different concentrations (1, 10, 100, and 500 μg/mL).

In terms of plumule and root length, with an example in Figure 5, the ‘Bidni’ acid-hydrolyzed extract at 100 μg/mL exhibited the longest plumule length (16.18 ± 6.70), while the shortest was observed in ‘Malti’ methanolic extract at 500 μg/mL (4.46 ± 1.35). For root length, ‘Bajda’ alkaline extract displayed the longest root length (57.72 ± 26.17), with ‘Malti’ methanol showing the shortest root length (10.75 ± 8.79). All measurements were expressed in mm. 

The Kruskal–Wallis ANOVA analysis shows no significant difference between cultivars in plumules treated with 500 (*p*-value = 0.198), 100 (*p*-value = 0.082), 10 (*p*-value = 0.260), and 1 μg/mL (*p*-value = 0.740) of extract, but there was a significant difference in treatments. Specifically, for plumule length treated with 500 (*p*-value = 0.004) and 100 (*p*-value = 0.004) μg/mL, significant differences were seen between alkaline- and acid-hydrolyzed extracts (*p*-value = 0.005 at 500 μg/mL; *p*-value = 0.007 at 100 μg/mL), as well as between methanolic and acid-hydrolyzed extracts (*p*-value = 0.041 at 500 μg/mL; *p*-value = 0.023 at 100 μg/mL). For lettuce seeds treated with both 500 μg/mL and 100 μg/mL of extract, the highest plumule length was found in acid hydrolysis (21.00 mm and 21.11 mm), while the lowest was found in alkaline hydrolysis (9.22 mm and 9.78 mm). Regarding the lettuce seeds treated with 10 μg/mL of extract, a significant difference (*p*-value = 0.003) was observed between alkaline and acid hydrolysis but not between cultivars (*p*-value = 0.200). The highest plumule length was found in acid hydrolysis (19.67 mm), while the lowest was found in alkaline hydrolysis (7.44 mm). No significant difference was found for the plumule length of lettuce seeds treated with 1 μg/mL of extract for both cultivars (*p*-value = 0.740) and treatment (*p*-value = 0.078). As for the root length of the lettuce seeds treated with 500 μg/mL, a significant difference (*p*-value = 0.003) was observed between ‘Malti’ and ‘Bidni’ (*p*-value = 0.021) extracts and between ‘Malti’ and ‘Bajda’ extracts (*p*-value = 0.019), with the highest root length in ‘Bidni’ extract (17.44 mm) and the lowest in ‘Malti’ (7.22 mm). For 100 μg/mL, a significant difference (*p*-value = 0.003) was observed between methanol- and acid-hydrolyzed extracts, with acid having the highest root length of 19.11 mm and methanol having the lowest root length of 8.44 mm. Still, no significant difference (*p*-value = 0.162) was observed between the cultivars. Additionally, there was no significant difference observed for both treatments (*p*-value = 0.740 for 10 μg/mL; *p*-value = 0.2751 μg/mL) and cultivar (*p*-value = 0.074 for 10 μg/mL; *p*-value = 0.095 1 μg/mL) for 10 and 1 μg/mL concentrations.

### 3.7. Brine Shrimp Lethality Assay 

Table 2 shows that as the concentrations of extracts increase, the percentage mortality rate of *Artemia salina* increases. However, it decreased again at lower concentrations. The lowest LD_50_ was found in ‘Bajda’ alkaline (2.2 ± 0.8 μg/mL) whilst the highest was found in ‘Bidni’ methanol (14.6 ± 6.6 μg/mL). The lethal concentration (LC_50_) for brine shrimp with a 95% confidence level was determined by Probit analysis shown in Supplementary Material Figure S9. 

### 3.8. HPLC Analysis

**Figure 6:**
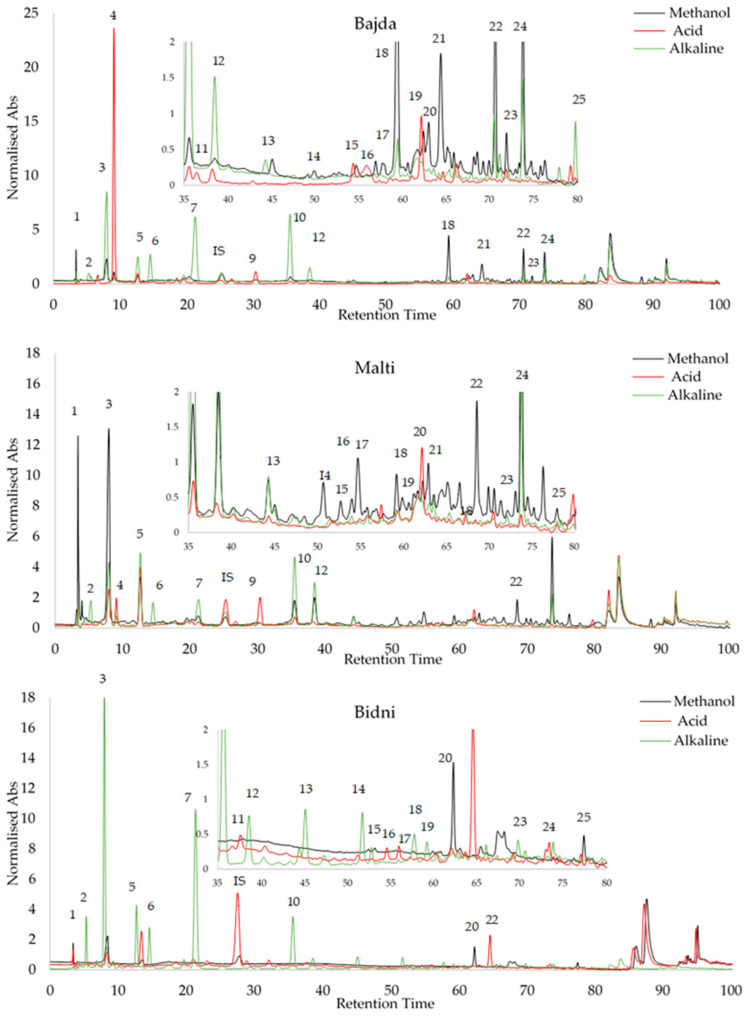
Peak identification of olive mill waste extracts via RRT analysis of external standards using syringic acid as internal standard.

Peak identification was accomplished by analyzing the relative retention time (RRT) of external standards, utilizing syringic acid as an internal standard, as detailed in Table S4. The standards exhibited a recovery rate ranging from 85.18% to 99.99%. Oleocein had the highest limit of detection (LOD) at 1.56 μg/mL, while oleochantal and vanillin had the lowest at 0.1 μg/mL. With regards to the limit of quantification (LOQ), catechin had the highest value at a concentration of 2.65 μg/mL, while vanillin had the lowest at 0.3 μg/mL. Subsequently, the three Maltese cultivars underwent the same HPLC procedure for all extraction methods, and peak identification was conducted, as seen in Figure 6. The retention times of the compounds on the three chromatograms are as follows: 1 = Gallic Acid, 2 = 3-Hydroxytyrosol, 3 = 2-(-4-Hydroxy Phenyl) Ethanol, 4 = 4-Hydroxy-Benzoic Acid, 5 = Catechin, 6 = vanillic acid, 7 = 2,4-Dihydroxy Benzoic Acid, 8 = Syringic Acid (IS), 9 = Vanillin, 10 = p-Coumaric Acid, 11 = 3-Methoxy-4-Hydroxy Cinnamic Acid, 12 = Ferulic Ac-id, 13 = 2-Hydroxy-Cinnamic Acid (trans), 14 = Ellagic Acid, 15 = Luteolin 7-Glucoside, 16 = Apigenin 7-Glucoside, 17 = Salicylic Acid, 18 = Oleacein, 19 = Oleuropein, 20 = Oleuoside, 21 = Oleocanthal, 22 = Ligstorside, 23 = 3′,4,5,7-Tetreahydroxy Flavone, 24 = Quercetin, and 25 = 5,7-Dihydroxy Flavone. Table 3 shows the HPLC profiling of OMW extracts with different treatments. Among the compounds identified, 3-hydroxytyrosol and oleacein were the most abundant, while gallic acid was found to be the least abundant. All data in Table 3 are expressed in μg/mL.

A Kruskal–Wallis ANOVA showed that in the HPLC analysis, from the 25 compounds tested, 17 compounds emerged as significantly influenced by the treatment, while the remaining compounds did not show any significant impact. Notably, 3,4,7 trihydroxyflavone (*p*-value = 0.034) and gallic acid (*p*-value = 0.004) exhibited a significant difference between the ‘Bidni’ and ‘Bajda’ cultivars. A significant difference (*p*-value = 0.005) was also observed in the presence of 5,7 dihydroxyflavone between the ‘Bidni’ and ‘Malti’ cultivars. For compounds such as quercetin (*p*-value < 0.005), 3,4,5,7 tetrahydroxyflavone (*p*-value = 0.021), ligstroside (*p*-value < 0.005), and trans-cinnamic acid (*p*-value < 0.005), significant differences were revealed between the ‘Malti’ and ‘Bidni’, as well as between the ‘Bidni’ and ‘Bajda’, cultivars. Oleuropein (*p*-value = 0.001), apigenin-7-glucoside (*p*-value = 0.025), and vanillic acid (*p*-value = 0.003) highlighted a significant difference between the ‘Malti’ and ‘Bajda’ cultivars in their response to the treatment. Lastly, Rutin (*p*-value = 0.010), 4,5,7 trihydroxyflavone (*p*-value = 0.039), salicylic acid (*p*-value = 0.039), isoquercetin (*p*-value = 0.035), 2,4-dihydroxybenzoic acid (*p*-value = 0.025), catechin (*p*-value = 0.027), and 3-0-methyl gallic acid (*p*-value = 0.028) were notably affected by the treatment, although no significant differences were observed between the cultivars for these compounds.

### 3.9. Multivariate Data Analysis 

The utilization of multivariate data analysis in the examination of chemical data is commonly referred to as chemometrics. This analytical approach proves advantageous when addressing extensive and intricate datasets characterized by numerous variables that may exhibit interdependencies. In the course of this investigation, Principal Component Analysis (PCA), two-way cluster analysis, and matrix correlations were employed to extract insights, identify patterns, and discern relationships present in the data.

#### 3.9.1. Principal Component Analysis (PCA)

**Figure 7:**
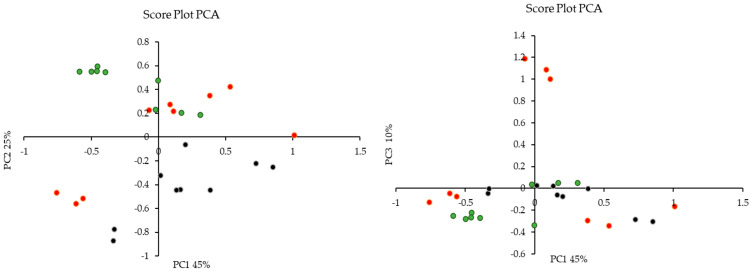
Principal Component Analysis (PCA) score plot revealing treatment-based clustering of olive mill waste were (•) is acid, (•) is alkaline and (•) is methanolic extraction.

Principal Component Analysis (PCA) was employed to discern potential outliers or clustering patterns within the dataset. Principal Component 1 (PC1) elucidated 45% of the total variation in the data, while PC2 and PC3 accounted for 25% and 10%, respectively. The cumulative explanatory power of the data was notably high for PC1, PC2, and PC3, encompassing approximately 80% of the dataset’s variation. Figure 7 shows the score plot, which demonstrated clustering based on the treatment of olive mill waste (methanol, acid, and alkaline) rather than the specific cultivar of origin (‘Bidni’, ‘Malti’, and ‘Bajda’).

**Figure 8:**
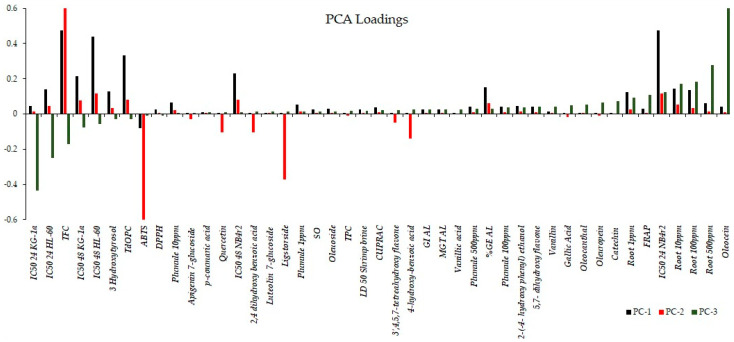
Rotated loadings depicting relationships after component rotation, aiding interpretation and simplifying structure, with stronger associations indicated by higher loadings.

The bar graph shown in Figure 8 illustrates the rotated loadings, which represent the loadings of the original variables after applying a rotation transformation to the principal components. The rotation is aimed at enhancing the interpretability of the components, creating a simpler and more meaningful structure. These loadings indicate the strength and direction of the relationship between each variable and each component. Higher loadings suggest a stronger association between a variable and a component, while loadings close to zero indicate a weaker or negligible relationship. For PC1, variables with the most substantial positive loadings encompass the bioactivity observed after 48 h, expressed as the IC_50_ for the cell lines KG-1a, NB4r2, and HL-60, in conjunction with the total flavonoid content and ortho diphenolic content of the extract. PC2 retains the same variables with the most significant positive loadings as PC1. However, in the case of PC2, the most negative loadings involve the IC_50_ against ABTS radical cations, as well as the concentrations of ligstorside, 4-hydroxy-benzoic acid, and 2,4-dihydroxy benzoic acid. For PC3, the most substantial positive loadings include the observed bioactivity on root growth and the concentration of oleacein in the extracts. Conversely, the most negative loadings in PC3 are associated with the bioactivity of the cell lines after 24 h of extract exposure.

#### 3.9.2. Two-Way Cluster Analysis 

The application of two-way cluster analysis, also known as biclustering or co-clustering, is a statistical technique used to simultaneously group observations (rows) and variables (columns) in a dataset. It aims to identify subsets of rows and columns that exhibit similar patterns or relationships, allowing for a more comprehensive understanding of the data. Figure S10 in the Supplementary Material illustrates the two-way cluster analysis using Ward’s method. This method aims to minimize the variance within clusters when merging them, resulting in compact and well-separated clusters from the obtained clustering; three major clusters were discerned. The first cluster comprised extracts derived from the ‘Bidni’ cultivar following methanolic and acid hydrolysis. This cluster exhibited notable characteristics, including a high concentration of total phenolic content, elevated ferric reduction potential, and a stimulatory effect on plume and root growth observed at 500 μg/mL. However, it demonstrated low bioactivity against HL-60 cell lines after 24 h of exposure. The second cluster encompassed a heterogeneous array of extracts derived from ‘Bajda’ and ‘Malti’ cultivars obtained through various extraction methods, with the exception of ‘Bajda’ extracts obtained after acid hydrolysis. Remarkably, these acid-derived ‘Bajda’ extracts clustered with ‘Bidni’ alkaline-hydrolyzed extracts, both of which formed the third cluster. In contrast to the second cluster, where characterization was less distinct, the third cluster, akin to the first cluster, was characterized by a heightened concentration of total phenolic content, increased ferric reduction potential, and a stimulatory effect on root growth observed at 500 μg/mL. In addition to these characteristics, ‘Bajda’ acid-derived extracts within the third cluster exhibited a high concentration of secoiridoid compounds, specifically oleuropein and oleacin, along with phenolic acids such as gallic acid, ellagic acid, 2,4-dihydroxybenzoic acid, 4-hydroxybenzoic acid, and 2-hydroxycinnamic acid, as well as catechin flavonoids. Moreover, the two-cluster analysis revealed a tendency for these compounds to cluster together, suggesting a shared biochemical synthetic pathway. Concerning ‘Bidni’ alkaline extracts, they manifested a higher concentration of secoroiod compounds, including oleocanthal and oleoside, along with their simple phenol-building monomers tyrosol and hydroxytyrosol, as well as phenolic acids, namely ferulic and vanillic acid.

#### 3.9.3. Matrix Correlation Analysis 

**Figure 9:**
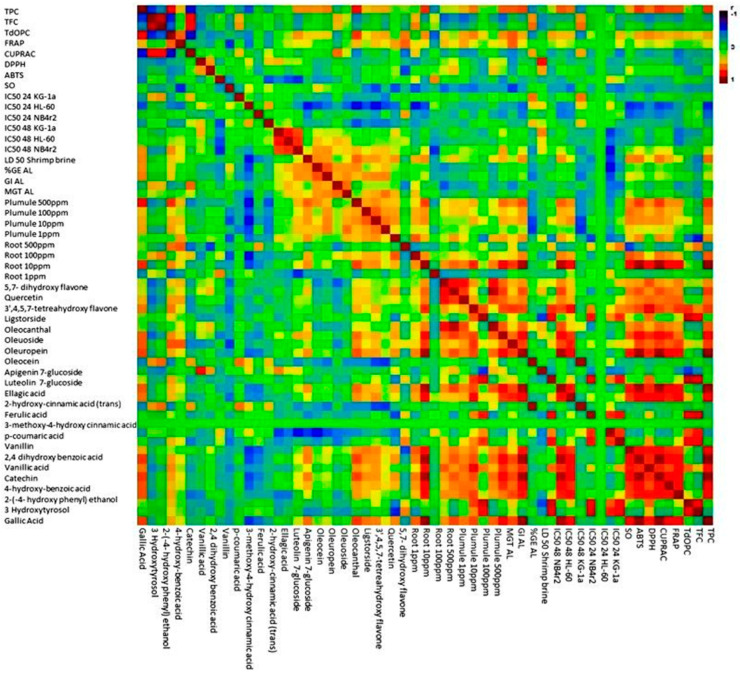
Matrix correlation analysis reveals relationships among phenolic compounds and bioactivity assays in olive mill waste.

In order to identify the relationship between the different variables, a matrix correlation analysis was carried out. The application of matrix analysis scrutinizes the relationships between multiple variables in a dataset. Through the application of matrix analysis, the correlation coefficient between pairs of variables is extracted and organized into a matrix format. The correlation matrix provides valuable insights into the strength and direction of linear associations between variables, enabling the identification of patterns and dependencies within the data. From Figure 9, the concentration of various phenolic acids, such as gallic acid, 4-hydroxybenzoic acid, 3-o-methlygallic acid, vanillic acid, 2,4 dihydroxybenzoic acid, vanillin, 2-hydroxycinnamic acid, ellagic acid, and 3,4 dimethoxycinnamic acid, exhibited significant positive correlations with one another. Additionally, specific positive correlations were observed between ferulic acid and p-coumaric acid, as well as between ferulic acid and 3-methoxy 4-hydroxycinnamic acid. In terms of simple phenols, namely tyrosol, hydroxytyrosol, oleocanthal, oleoside, oleacein, oleuropein, and ligstroside, there were strong positive significant correlations observed among them. Furthermore, these simple phenols also displayed positive correlations with other phenolic acids, such as ferulic acid, p-coumaric acid, and 3-methoxy 4-hydroxy cinnamic acid. Additionally, these simple phenols exhibited positive correlations with flavonoid compounds, including 5,7-dihydroxyflavone, 3,4,5,7 tetrahydroxyflavone, 4′,5,7-trihydroxyflavone and querectin. In relation to the various bioactivity assays conducted, a majority of the bioactivity was exhibited against cancer cell lines. A notable inverse relationship with the concentration of certain seciroiod compounds, specifically oleacein, oleuropein, as well as simple phenolic acids such as gallic acid, ellagic acid, vanillic acid, trans-cinnamic, 3,4 dimethoxycinnamic acid, and 2,4 dihydroxybenzoic acid was observed. Regarding flavonoids, the concentrations of 3,5,7 trihydroxyflavone and 5,7 dihydroxyflavone displayed a significantly negative correlation with the observed bioactivity against cancer cell lines. The investigation into toxicity utilizing the shrimp brine model revealed a noteworthy inverse relationship with the concentration of distinct phenols, specifically tyrosol, hydroxytyrosol, 4-hydroxybenzoic acid, 3-methoxy-4-hydroxycinnamic acid, and p-coumaric acid. An investigation into the allopathic behavior revealed a stimulating impact on root growth, which exhibited a positive correlation with the levels of vanillin, vanillic acid, 2,4 dihydroxybenzoic acid, 3-o-methly gallate, 4-hydroxybenzoic acid, ellagic acid, trans-cinnamic acid, and 3,4 dimethoxycinnamic acid. Conversely, the concentrations of 3-methoxy-4-hydroxycinnamic acid, ferulic acid, and flavonoid compounds, specifically quercetin, 4,5,7 trihydroxflavone, and 3,4,5,7 tetrahydroxyflavone, were found to have a detrimental effect on plume growth.

## 4. Discussion

### 4.1. Non-Adherent Cell Lines

The MTT assay was employed to analyze cytotoxicity across three distinct cell lines. This assay is used to measure cellular metabolic activity as an indicator of cell viability, cytotoxicity, and proliferation. For the results obtained, the three cultivars extracted with different treatments showed that NB4r2 has the lowest IC_50_s, followed by KG-1A and HL-60. Notably, HL-60 cell lines underwent 83 passages, which could lead to alternations in morphology, growth rate, response to stimuli, transfection efficiency, and protein expressions, as mentioned by Anderle et al. Consequently, this may account for the higher IC_50_ values observed in the HL-60 cell lines. [49]. 

The three exposure times led to a substantial reduction in the proliferation rate across the three cell lines. This indicates that the extracts exerted a mitigating effect on cell proliferation, as evidenced by the decreased division of cells, thereby reducing the progression of leukemic cancer. Specifically, for HL-60 cell lines, ‘Malti’ acid, ‘Bajda’ alkaline, and ‘Bidni’ alkaline-hydrolyzed extracts demonstrated lower IC_50_ values after 24 h of exposure. Additionally, ‘Malti’ methanol was found to be most effective for KG-1a cells at 24 h, which is advantageous as it implies efficacy at a shorter exposure time. Furthermore, the majority of the tested extracts displayed lower IC_50_ values after 72 h of exposure on the cells, likely due to the cells being more saturated with the extract. This is common and can be found in the literature, as mentioned by Tajudin et al. [50]. In fact, it was shown that in the MTT assay conducted, the cell proliferation rate was significantly reduced with concentration dependence, especially for the first 24 h, and it was further reduced at 48 and 72 h of exposure [50].

### 4.2. Staining of Cells

Three distinct staining procedures were employed, including Giemsa staining, hematoxylin and eosin staining, and quinacrine dihydrochloride staining. At a concentration of 10 ppm, the majority of cells exhibited well-defined cell walls and nuclei with no chromatin condensation. However, with an increase to 100 ppm, cells began to exhibit signs of apoptosis, such as cell shrinkage and the release of apoptotic bodies, indicating a concentration-dependent effect. Specifically, apoptotic cells showed reduced size and chromatin condensation, leading to increased fluorescence upon staining with quinacrine dihydrochloride. Additionally, quinacrine dihydrochloride staining revealed the formation of membrane vesicles containing intensely stained chromatin, which originated from the plasma membrane and eventually separated to form apoptotic bodies. While most cells exhibited typical morphological changes indicative of apoptosis, the possibility of necrosis in some cells during treatment cannot be entirely ruled out. This was corroborated by caspase-3 activation and apoptotic regulation assays.

### 4.3. DNA Laddering 

DNA laddering, a distinctive fragmentation pattern observed during apoptosis, represents a hallmark of programmed cell death [51]. Analysis reveals an increased incidence of bends at higher concentrations, suggesting a heightened influence of apoptotic inducers on cellular processes, leading to the enhanced activation of apoptotic pathways and subsequent DNA fragmentation. This is seen in studies by Nousis et al. and Fabiani et al., demonstrating DNA damage induced by olive mill wastewater and olive oil extracts rich in biophenols at higher concentrations [18,52]. Moreover, higher concentrations may prompt a greater proportion of cells to undergo apoptosis concurrently, amplifying overall DNA fragmentation and the presence of bends in the ladder pattern [53,54].

### 4.4. Caspase-3 Activation

Caspase-3, an essential protease enzyme in the execution phase of apoptosis, represents an integral component of the programmed cell death mechanism [55]. As a constituent of the caspase protein family, it functions as a cysteine protease, selectively cleaving target proteins at specific aspartic acid residues [56,57]. The activation of caspase-3 during apoptosis involves proteolytic cleavage, yielding its active form, which subsequently catalyzes the cleavage of diverse cellular proteins, eliciting characteristic morphological and biochemical alterations associated with apoptosis, such as DNA fragmentation and cell shrinkage [58]. This was evidenced in cell staining analysis (Figure 2), revealing prominent chromatin condensation and cell shrinkage predominantly at higher concentrations. Notably, in Section 3.4, it can be seen that ‘Bidni’ exhibited the highest caspase-3 activity at both concentrations, indicating augmented apoptotic activity within KG-1a cells. Elevated caspase-3 levels signify active apoptosis, potentially triggered by various factors, including exposure to apoptotic stimuli [59] and cellular stress [60].

### 4.5. Apoptotic Regulation

With regards to BAX and BCL-2, BCL-2 functions as an anti-apoptotic protein, inhibiting cell death by preventing the release of cytochrome c from mitochondria and the subsequent activation of caspases [61]. Therefore, the examination of Section 3.5 reveals that decreased BCL-2 levels compared to the control may suggest compromised anti-apoptotic defenses, thereby facilitating the initiation of apoptosis, as evidenced by Shinoura et al. [62]. Conversely, BAX serves as a pro-apoptotic protein, promoting cell death via mitochondrial outer membrane permeabilization (MOMP) and subsequent cytochrome c release and caspase activation [63]. Elevated BAX levels compared to the control, as demonstrated in Section 3.5, may signify heightened pro-apoptotic signaling, facilitating mitochondrial dysfunction and apoptotic cell demise. Studies by Wood et al. [64], Wei et al. [65], Gross et al. [66], and Selvakumaran et al. [67] collectively support the concept of increased BAX levels driving pro-apoptotic signaling and subsequent apoptotic cell death, with Wei et al. [65] emphasizing BAX’s role in initiating mitochondrial dysfunction and cell death.

### 4.6. Effect of Seed Germination

The majority of allelochemicals in OMW are mainly phenolic compounds [68]. Phenolics are well known to interfere with several physiological processes associated with seed germination as well as plant growth and development [69]. The present results indicated that OMW extracts, particularly at higher concentrations, notably reduced the germination of lettuce seeds. Specifically, the ‘Malti’ extract showed the lowest germination rate, impacting the seed’s growth rate significantly, whereas the ‘Bajda’ extract exhibited a higher percentage of germination, suggesting a comparatively lesser impact on the seeds compared to the ‘Malti’ extract. These results align with a study conducted by Saleh, in which it was found that the allelopathic potential of olive processing waste on maize varied depending on the concentration of the extract [70]. 

#### Root and Plumule Length

Olive mill waste can impact the plumule and root development of lettuce seeds. The specific effects depend on factors such as the concentration and composition of the waste, as well as the duration of exposure. Generally, olive mill waste contains organic compounds, such as phenols and polyphenols, which can have allelopathic effects on other plants. These compounds may inhibit seed germination, root elongation, and overall seedling growth [2]. Additionally, the high organic content in olive mill waste can alter soil properties, affecting nutrient availability and soil structure, which, in turn, may influence seedling development [71]. From the results obtained, the analysis of the effects of ‘Bidni’ extracts on plumule length indicates that higher concentrations exhibit greater effectiveness on lettuce seeds, as evidenced by longer plumules. This suggests that higher concentrations of this extract exert stronger allelopathic effects, which may include inhibiting seed germination, root elongation, and overall seedling growth. Conversely, lower concentrations had a minimal effect on lettuce seeds. It was also evident that ‘Bidni’ extract treated with lower concentrations resulted in longer roots compared to higher concentrations. It is possible that lower concentrations represent an optimal range where the allelopathic effects of the olive mill waste extract are minimized, allowing for improved seed germination and seedling growth. The examination of ‘Bajda’ and ‘Malti’ extracts on plumule length reveals that at higher concentrations, the plumule length was shorter. This could be attributed to the elevated concentration of allelopathic compounds present in olive mill waste extracts. Higher concentrations possibly hindered cell division and elongation in the plumule, consequently leading to reduced length. Observing the root length, it was apparent that lower concentrations resulted in longer roots, likely for the same reasons observed with the ‘Bidni’ extracts.

Research on the utilization of OMW in lettuce seed production yielded diverse outcomes. Kelepesi et al. discovered that the addition of OMW into the growth medium improved seed emergence for chicory and specific lettuce varieties but led to reduced leaf weight and quantity [72]. Conversely, Naman et al. reported that raw OMW had an inhibitory effect on lettuce germination and growth, while treated OMW, particularly when diluted, stimulated growth [73]. Furthermore, Ouzounidou et al. found that biotreated OMW, especially with added proline, moderated the adverse effects of OMW on lettuce growth [74]. These studies collectively suggest that the effect of OMW on lettuce seeds in an allelopathic assay may be complex and dependent on various factors.

### 4.7. Shrimp Brine Assay 

The brine lethality assay using OMW extracts exhibited considerable cytotoxic effects. Results reveal that OMW extracts have a positive lethal effect on the larvae of brine shrimp after 24 h. This indicates that the extracts are biologically active, with mortality percentages gradually increasing with the rise in extract concentration, highlighting a concentration-dependent effect. Similar to the results obtained, Gueboudji et al. revealed that OMW extracts showed a positive lethal effect on *Artemia Salina* after 24 h of exposure [26]. 

One indicator of the toxicity of a substance is LD_50_, which refers to the amount (i.e., lethal dose or concentration) of a substance that kills 50% of the test organisms [75]. Results found in Table 2 showed LD_50_ values ranging from 0.0022 mg/mL to 0.0146 mg/mL. According to Geran et al., activities are significant if LD_50_ is below 0.03 mg/mL [76]. Thus, the tested extracts demonstrate considerable potency in this assay. A Kruskal–Wallis ANOVA analysis of LD_50_ indicated no significant difference between cultivars. However, a significant difference was observed between alkaline and methanolic extracts, with alkaline hydrolysis yielding the lowest LD_50_ median of 7.56, while methanolic extraction produced the highest LD_50_ median of 18.78.

### 4.8. OMW HPLC Profiling

For the identification of olive mill waste extracts, a total of 25 compounds were successfully identified and quantified with regard to methanolic and hydrolyzed extracts. From these results, it can be clearly seen the 3-hydroxytyrosol and oleacein were the predominant compounds. Oleacein, which is a potent antioxidant, was found in various parts of the olive tree, including the leaves and fruits [77,78,79]. However, in the previous literature, there is no specific mention of its presence in olive mill waste extracts. These two compounds were notably present in the highest concentrations across all hydrolyzed and methanolic extracts, except for ‘Malti’ acid and ‘Bidni’ acid. ‘Bidni’ alkaline extract recorded the highest concentration of 3-hydroxytyrosol at 188.82 ± 0.34 μg/mL. Comparatively, ‘Bidni’ acid and methanol, as well as ‘Malti’ acid extracts, showed fewer compounds in contrast to the other extracts. However, the ‘Bajda’ acid extract exhibited a higher compound content compared to the other extraction methods. This observation was corroborated in cell culture experiments, where the ‘Bajda’ acid extract demonstrated the most effectiveness. This suggests a potential correlation between the compound’s diversity within the extract and its biological activity.

In the literature, a range of compounds was identified in methanolic olive mill waste extracts, including hydroxytyrosol glucoside, hydroxytyrosol, tyrosol, verbascoside, and a derivative of oleuropein [80,81,82]. The acid and alkaline hydrolysis of olive mill waste extracts were found to yield residual oil with a fatty acid profile similar to virgin olive oil, as well as high levels of phenolic compounds such as tyrosol and hydroxytyrosol [83]. Corresponding to the findings, hydroxytyrosol emerges as the principal phenolic compound in these extracts [84]. With regards to oleacein, it is a potent antioxidant found in various parts of the olive tree, such as the leaves and fruits [80,81]. 

## 5. Conclusions

In conclusion, the bioactive potential of olive mill waste (OMW) obtained from cultivars grown in the Maltese Islands was investigated across various assays. Using the MTT assay for cytotoxicity analysis, concentration-dependent effects on non-adherent cell lines were observed, with distinct IC_50′_s values varying among different cultivars. The extracts showcased a mitigating effect on cell proliferation, particularly noticeable after 24 and 72 h of exposure. Moreover, staining procedures further affirmed concentration-dependent cellular changes, with higher concentrations inducing chromatin condensation and apoptotic body formation. The DNA laddering analysis unveiled enhanced DNA fragmentation at higher concentrations, suggesting the activation of apoptotic pathways. Furthermore, the caspase-3 activation assays provided additional support, revealing elevated protease activity indicative of apoptotic induction by the extracts. The evaluation of apoptotic regulators BAX and BCL-2 hinted at a shift towards pro-apoptotic signaling at higher concentrations. With regards to the seed germination assays, it disclosed the allelopathic effects of OMW extracts on lettuce seeds, affecting germination rates and seedling growth. Moreover, the brine lethality assay demonstrated substantial cytotoxic effects, with concentration-dependent lethality observed on brine shrimp larvae. Lastly, the HPLC profiling identified 3-hydroxytyrosol and oleacein as predominant compounds in the extracts, potentially linked to their biological activity. Overall, these findings highlight the diverse bioactive potential of OMW from Maltese cultivars, suggesting its versatility in various applications.

## Figures and Tables

**Table 1:** Allelopathic assay results for each olive mill waste extract at a concentration of 500, 100, 10, and 1 μg/mL. The following table shows the germination rate (%), germination energy (%), germination index, plumule length (mm), root length (mm), and ratio.

Allelopathy							
Sample	Concentration (μg/mL)	Germination Rate (%)	Germination Energy (%)	Germination Index	Plumule (mm)	Root (mm)	Ratio
Control	/	90.00 ± 0.36	43.33 ± 2.87	13.72 ± 0.35	14.20 ± 4.0	40.95 ± 8.72	3.01 ± 0.79
Bidni Methanol	500	72.08 ± 6.29	50.00 ± 13.23	8.31 ± 1.93	13.41 ± 3.66	35.84 ± 9.04	2.86 ± 1.00
	100	84.17 ± 2.59	66.67 ± 2.89	10.44 ± 0.93	12.90 ± 2.29	32.57 ± 8.23	2.61 ± 0.84
	10	84.38 ± 2.61	73.33 ± 2.89	10.38 ± 1.40	10.40 ± 1.65	37.22 ± 7.69	3.63 ± 0.90
	1	84.58 ± 2.77	68.33 ± 17.56	10.69 ± 2.15	10.34 ± 3.80	31.76 ± 7.08	3.45 ± 1.41
Bidni Acid	500	64.47 ± 2.28	11.67 ± 2.89	6.13 ± 0.72	13.72 ± 7.44	31.80 ± 19.97	2.30 ± 0.99
	100	81.67 ± 2.04	56.67 ± 7.64	9.80 ± 0.64	16.18 ± 6.70	37.39 ± 20.25	2.38 ± 1.12
	10	86.25 ± 1.66	71.67 ± 11.55	10.73 ± 0.74	13.58 ± 4.34	26.06 ± 8.52	2.14 ± 1.06
	1	86.67 ± 1.98	80.00 ± 10.00	10.78 ± 1.30	9.97 ± 3.65	33.55 ± 8.38	3.83 ± 1.83
Bidni Alkaline	500	70.21 ± 3.16	21.67 ± 16.07	7.65 ± 0.50	10.12 ± 2.01	29.32 ± 11.15	5.75 ± 4.39
	100	90.21 ± 1.51	51.67 ± 7.64	13.23 ± 1.50	11.58 ± 4.13	41.08 ± 17.72	3.72 ± 1.54
	10	95.42 ± 0.81	23.33 ± 7.64	16.78 ± 1.08	10.31 ± 4.06	54.04 ± 13.47	5.61 ± 1.49
	1	93.75 ± 1.30	23.33 ± 12.58	16.06 ± 1.54	9.89 ± 3.98	41.19 ± 19.36	4.90 ± 3.33
Bajda Methanol	500	69.79 ± 2.85	31.67 ± 7.64	8.15 ± 1.24	5.00 ± 0.84	16.26 ± 6.68	3.26 ± 1.22
	100	81.46 ± 3.30	56.67 ± 11.55	10.92 ± 0.61	9.47 ± 4.12	36.86 ± 12.52	4.46 ± 2.05
	10	82.71 ± 3.12	55.00 ± 8.66	11.00 ± 1.46	11.47 ± 2.68	35.00 ± 10.49	3.11 ± 0.89
	1	86.04 ± 1.15	65.00 ± 10.00	11.08 ± 0.13	9.64 ± 3.74	30.83 ± 6.93	3.54 ± 1.25
Bajda Acid	500	59.58 ± 6.93	11.67 ± 7.64	5.76 ± 1.35	8.15 ± 1.63	45.38 ± 14.27	5.69 ± 1.81
	100	76.88 ± 7.26	58.33 ± 15.28	9.79 ± 2.46	9.85 ± 4.49	46.60 ± 16.25	5.44 ± 2.31
	10	83.54 ± 1.87	53.33 ± 10.41	10.95 ± 1.04	7.88 ± 2.09	42.56 ± 9.37	5.79 ± 2.19
	1	84.79 ± 2.76	50.00 ± 15.00	11.77 ± 1.04	7.58 ± 2.69	29.87 ± 11.78	4.29 ± 2.22
Bajda Alkaline	500	49.79 ± 3.41	3.33 ± 2.89	4.92 ± 1.11	5.03 ± 2.77	33.79 ± 23.61	8.25 ± 8.97
	100	86.04 ± 3.05	36.67 ± 12.58	12.90 ± 1.46	8.57 ± 4.05	57.72 ± 26.17	7.16 ± 2.95
	10	93.75 ± 2.46	31.67 ± 16.07	15.87 ± 2.68	7.47 ± 2.38	41.53 ± 9.83	6.00 ± 1.96
	1	94.38 ± 2.00	28.33 ± 18.93	16.41 ± 1.42	11.95 ± 4.43	43.35 ± 13.30	4.02 ± 1.49
Malti Methanol	500	48.75 ± 2.45	18.33 ± 2.89	4.87 ± 0.49	4.46 ± 1.35	10.75 ± 8.79	2.26 ± 1.58
	100	71.04 ± 4.56	41.67 ± 7.64	8.22 ± 1.49	7.79 ± 3.42	39.28 ± 19.77	5.29 ± 3.02
	10	84.79 ± 3.85	60.00 ± 13.23	11.46 ± 2.24	15.01 ± 8.33	29.63 ± 12.66	2.46 ± 1.69
	1	85.42 ± 0.79	66.67 ± 2.89	10.53 ± 0.93	10.11 ± 2.08	25.34 ± 5.11	2.62 ± 0.80
Malti Acid	500	61.04 ± 3.28	26.67 ± 18.93	6.07 ± 0.98	11.44 ± 4.20	22.94 ± 11.17	2.08 ± 0.86
	100	79.17 ± 3.41	61.67 ± 12.58	9.38 ± 1.51	12.64 ± 3.51	41.24 ± 14.60	3.41 ± 1.24
	10	86.88 ± 1.82	68.33 ± 7.64	11.44 ± 1.17	17.32 ± 6.24	41.29 ± 10.40	2.81 ± 1.53
	1	85.21 ± 1.27	73.33 ± 12.58	10.87 ± 0.14	13.33 ± 4.79	36.02 ± 11.14	2.89 ± 1.14
Malti Alkaline	500	26.67 ± 5.68	0.01 ± 0.00	2.32 ± 0.90	4.97 ± 1.98	13.57 ± 8.12	3.22 ± 1.96
	100	75.83 ± 1.69	38.33 ± 5.77	8.64 ± 0.67	8.12 ± 3.95	43.88 ± 19.57	5.97 ± 2.90
	10	88.75 ± 2.31	35.00 ± 13.23	13.64 ± 1.14	12.13 ± 2.17	46.26 ± 9.64	3.98 ± 1.34
	1	92.71 ± 1.49	38.33 ± 5.77	15.01 ± 1.29	12.12 ± 3.23	30.79 ± 8.67	2.70 ± 1.05

**Table 2:** Percentage mortality of shrimp Nauplii using olive mill waste extract at 500, 50, 10, and 0.1 μg/mL. The following table shows the number of surviving nauplii after 24 h of exposure, total number of survivors nauplii, percentage mortality, and LD_50_ in μg/mL.

% Mortality of Shrimp Nauplii							
Sample	Concentration (μg/mL)	Number of Surviving Nauplii (After 24 h)			Total Number of Nauplius Survivors	% Mortality	LD_50_ (μg/mL)
		T1	T2	T3			
Bidni Methanol	500	2	3	3	8	73.9 ± 6.8	14. 6 ± 6.6
	50	6	4	3	13	59.7 ± 12.7	
	10	4	5	4	13	56.7 ± 5.8	
	0.1	7	8	8	23	25.5 ± 9.4	
Bidni Acid	500	0	0	0	0	100.0 ± 0.0	2.9 ± 1.6
	50	2	3	3	8	74.2 ± 5.2	
	10	4	5	5	14	53.4 ± 3.0	
	0.1	7	8	6	21	32.1 ± 10.7	
Bidni Alkaline	500	0	0	0	0	100.0 ± 0.0	3.6 ± 1.6
	50	2	2	4	8	75.2 ± 10.0	
	10	6	7	6	19	38.5 ± 7.8	
	0.1	6	8	6	20	28.1 ± 15.1	
Bajda Methanol	500	3	2	2	7	76.7 ± 5.8	13.0 ± 1.3
	50	3	3	3	9	70.0 ± 0.0	
	10	4	6	4	14	51.9 ± 10.5	
	0.1	7	8	8	23	25.8 ± 5.2	
Bajda Acid	500	0	0	0	0	100.0 ± 0.0	4.4 ± 0.6
	50	3	5	4	12	60.0 ± 10.0	
	10	8	6	7	21	34.5 ± 6.6	
	0.1	7	7	8	22	26.7 ± 5.8	
Bajda Alkaline	500	0	1	0	1	96.7 ± 5.8	2.2 ± 0.8
	50	2	1	1	4	87.3 ± 4.7	
	10	3	4	3	10	68.8 ± 4.7	
	0.1	7	5	7	19	36.7 ± 11.5	
Malti Methanol	500	1	0	0	1	97.2 ± 4.8	3.8 ± 1.6
	50	4	3	2	9	72.9 ± 8.4	
	10	5	4	6	15	52.7 ± 11.9	
	0.1	5	8	8	21	25.3 ± 4.6	
Malti Acid	500	1	0	0	1	96.7 ± 5.8	6.4 ± 4.6
	50	5	6	5	16	46.7 ± 5.8	
	10	4	5	6	15	42.9 ± 15.9	
	0.1	8	6	5	19	36.7 ± 15.3	
Malti Alkaline	500	0	0	0	0	100.0 ± 0.0	2.7 ± 1.6
	50	2	1	0	3	90.6 ± 9.1	
	10	6	7	6	19	36.7 ± 5.8	
	0.1	6	8	6	20	35.2 ± 13.4	

**Table 3:** HPLC profiling of ‘Malti’, ‘Bidni’, and ‘Bajda’ extracts using 25 different standards. <LOD = value lower than LOD, >LOD–<LOQ = value falls between LOD and LOQ, meaning that the compounds can be detected but cannot be equally quantified; ND = Not detected. All data are in μg/mL.

	Compound	RRT	Malti Methanol	Malti Acid	Malti Alkaline	Bajda Methanol	Bajda Acid	Bajda Alkaline	Bidni Methanol	Bidni Acid	Bidni Alkaline
25	5,7-dihydroxy flavone	3.78	10.091 ± 0.048	9.631 ± 0.060	9.678 ± 0.044	<LOD	9.955 ± 0.164	6.916 ± 0.547	1.641 ± 0.030	< LOD	< LOD
24	Quercetin	3.05	2.225 ± 0.005	<LOD	<LOD	2.064 ± 0.001	2.032 ± 0.007	3.003 ± 0.018	<LOD	<LOD	2.157 ± 0.005
23	3′,4,5,7-tetreahydroxy flavone	2.96	3.572 ± 0.003	<LOD	<LOD	3.551 ± 0.001	3.782 ± 0.186	3.791 ± 0.002	<LOD	<LOD	<LOD
22	Ligstorside	2.92	1.002 ± 0.011	<LOD	<LOD	1.303 ± 0.013	2.254 ± 0.540	3.010 ± 0.011	0.286 ± 0.006	<LOD	1.619 ± 0.014
21	Oleocanthal	2.94	1.359 ± 0.022	ND	2.028 ± 0.089	1.284 ± 0.069	7.070 ± 0.235	<LOD	<LOD	<LOD	11.713 ± 0.368
20	Oleuoside	2.85	5.089 ± 0.046	5.081 ± 0.049	1.220 ± 0.034	<LOD	3.563 ± 0.622	1.668 ± 0.040	<LOD	1.990 ± 0.066	19.292 ± 0.122
19	Oleuropein	2.69	0.858 ± 0.010	<LOD	<LOD	6.265 ± 0.042	8.916 ± 0.837	1.137 ± 0.031	2.398 ± 0.240	<LOD	3.823 ± 0.021
18	Oleacein	2.54	13.518 ± 0.980	<LOD	12.199 ± 0.269	11.085 ± 0.021	18.345 ± 0.054	3.816 ± 0.027	5.651 ± 0.126	<LOD	9.828 ± 0.038
17	Salicylic acid	2.44	2.590 ± 0.019	<LOD	<LOD	<LOD	<LOD	<LOD	<LOD	<LOD	<LOD
16	Apigenin 7-glucoside	2.41	3.282 ± 0.009	<LOD	>LOD–< LOQ	8.109 ± 0.178	1.518 ± 0.036	2.145 ± 0.010	3.343 ± 0.129	>LOD–< LOQ	<LOD
15	Luteolin 7-glucoside	2.34	<LOD	>LOD–< LOQ	<LOD	>LOD–< LOQ	1.735 ± 0.066	>LOD–< LOQ	<LOD	<LOD	12.849 ± 0.046
14	Ellagic acid	2.22	0.699 ± 0.024	<LOD	<LOD	0.218 ± 0.001	1.971 ± 0.021	<LOD	<LOD	<LOD	>LOD–< LOQ
13	2-hydroxy-cinnamic acid (trans)	2.19	>LOD–< LOQ	>LOD–< LOQ	>LOD–< LOQ	>LOD–< LOQ	>LOD–< LOQ	>LOD -LOQ	>LOD–< LOQ	>LOD–< LOQ	>LOD–< LOQ
12	Ferulic acid	1.91	<LOD	<LOD	>LOD–< LOQ	<LOD	<LOD	<LOD	<LOD	<LOD	2.394 ± 0.008
11	3-methoxy-4-hydroxy cinnamic acid	1.78	1.055 ± 0.009	<LOD	0.974 ± 0.033	<LOD	ND	>LOD -LOQ	<LOD	<LOD	1.436 ± 0.068
10	p-coumaric acid	1.44	4.724 ± 0.042	2.188 ± 0.011	6.219 ± 0.018	2.348 ± 0.013	3.391 ± 0.018	11.358 ± 0.046	1.927 ± 0.007	2.067 ± 0.010	22.046 ± 0.056
9	Vanillin	1.22	4.103 ± 0.001	4.851 ± 0.007	4.106 ± 0.002	4.084 ± 0.001	7.641 ± 0.027	4.205 ± 0.012	<LOD	4.194 ± 0.005	4.655 ± 0.004
7	2,4 dihydroxy benzoic acid	0.82	<LOD	<LOD	<LOD	<LOD	1.900 ± 0.005	<LOD	<LOD	<LOD	<LOD
6	Vanillic acid	0.78	1.688 ± 0.302	1.191 ± 0.010	1.570 ± 0.004	2.011 ± 0.013	4.153 ± 0.027	2.221 ± 0.023	<LOD	1.544 ± 0.002	5.513 ± 0.184
5	Catechin	0.66	<LOD	<LOD	<LOD	<LOD	10.332 ± 0.277	<LOD	<LOD	<LOD	<LOD
4	4-hydroxy-benzoic acid	0.54	2.395 ± 0.009	<LOD	2.359 ± 0.004	<LOD	3.980 ± 0.731	2.458 ± 0.042	<LOD	2.317 ± 0.001	2.882 ± 0.011
3	2-(-4-hydroxy phenyl) ethanol	0.51	11.159 ± 0.075	8.482 ± 0.011	11.305 ± 0.011	3.763 ± 0.010	8.959 ± 0.808	9.431 ± 0.124	3.608 ± 0.019	7.321 ± 0.081	43.837 ± 0.105
2	3 Hydroxytyrosol	0.31	48.084 ± 0.035	4.699 ± 0.075	10.559 ± 0.032	6.034 ± 0.044	1.050 ± 0.063	32.838 ± 0.123	6.801 ± 0.312	2.565 ± 0.048	188.819 ± 0.341
1	Gallic Acid	0.26	0.480 ± 0.001	0.676 ± 0.008	0.785 ± 0.006	0.505 ± 0.006	7.136 ± 0.030	0.792 ± 0.053	<LOD	0.494 ± 0.001	0.484 ± 0.004

## Data Availability

The original contributions presented in the study are included in the article/Supplementary Material, further inquiries can be directed to the corresponding author.

## Supplementary Materials

The following supporting information can be downloaded at: https://www.mdpi.com/article/10.3390/foods13081152/s1, Figure S1: Calibration curve for bovine serum albumin for protein content., Figure S2: Calibration curve for BCL-2., Figure S3: Calibration curve for BAX., Table S1: Standards used for the identification of phenolic compounds in olive mill waste extracts., Figure S4: IC50’s of (A) NB4r2, (B) HL-60 and (C) KG-1a cell lines treated with olive mill waste extracts at 24 (black), 48 (red) and 72 (green) h of exposure., Figure S5: The percentage cell viability against concentration graph of ‘Bajda’ cultivar acid (red), methanol (black) and alkaline (green) extraction on NB4r2 cell line at 48 h of exposure., Figure S6: Caspase-3 activity on KG-1a cell lines after 24 h of exposure using different concentrations (100 and 10 ppm) for ‘Bidni’ (black), ‘Bajda’ (red) and ‘Malti’ (green) extracts with the addition of a control., Figure S7: BCL-2 content on KG-1a cell lines after 24 h (A) and 48 h (B) of exposure using different concentrations for ‘Bidni’ (black), ‘Bajda’ (red) and ‘Malti’ (green) extracts with the addition of a control., Figure S8: BAX content on KG-1a cell lines after 24 h (A) and 48 h (B) of exposure using different concentrations for ‘Bidni’ (black), ‘Bajda’ (red) and ‘Malti’ (green) extracts with the addition of a control., Figure S9: The percentage mortality probit of olive mill waste extracts at different extraction procedures., Figure S10: Two-way cluster analysis, utilizing Ward’s method, reveals three distinct clusters with ‘Bidni’ extracts dominating the first cluster’s biochemical profile.
