# Supplementary material for: Bioactive Potential of Olive Mill Waste Obtained from Cultivars Grown in the Island of Malta
Source: Foods. 2024 Apr 10;13(8):1152. doi: 10.3390/foods13081152 (PMC11049450; doi:10.3390/foods13081152)
Supplement: Supplementary file 1 [file foods-13-01152-s001.zip › foods-2929639-supplementary.pdf]

Supplementary material

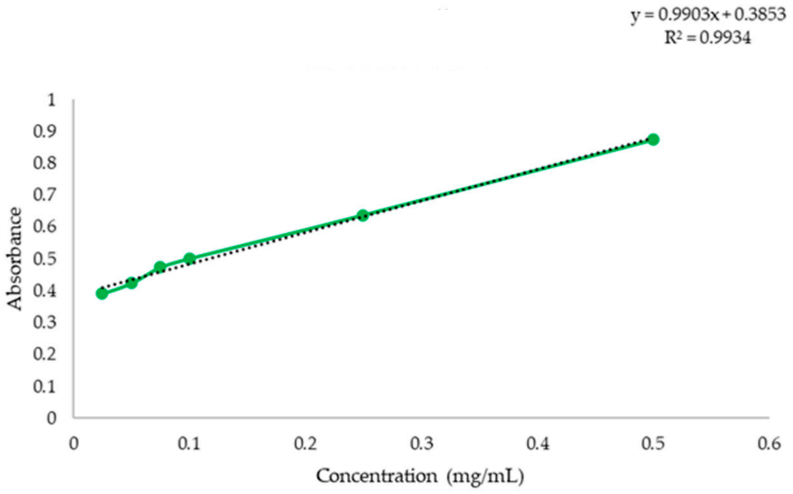


**Figure S1: Calibration curve for bovine serum albumin for protein content.**

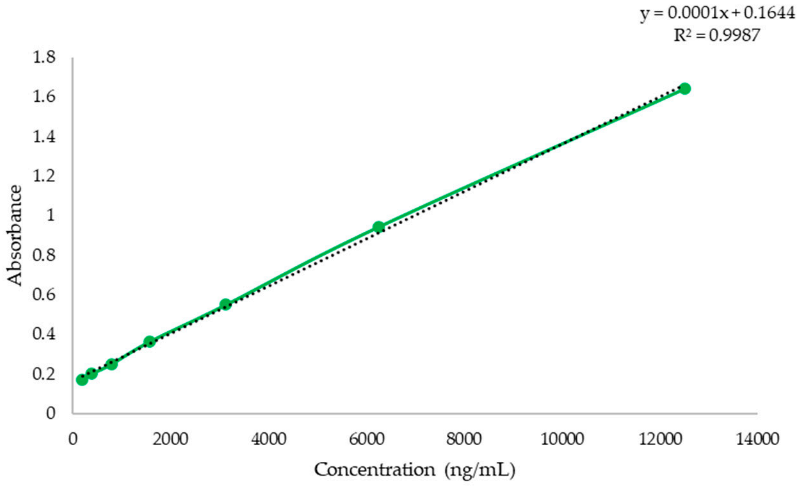


**Figure S2: Calibration curve for BCL-2.**

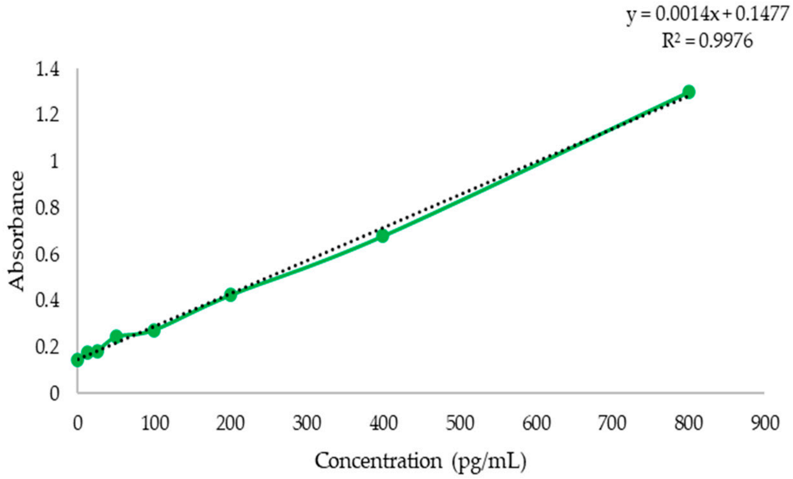


**Figure S3: Calibration curve for BAX.**

**Table S1: Standards used for the identification of phenolic compounds in olive mill waste extracts.**

| Standards | | | | | | | |
|---|---|---|---|---|---|---|---|
| Compound | RRT | Regression Equation | $R^2$ | Range µg/ml | LOD µg/ml | LOQ µg/ml | % Recovery |
| 5,7- dihydroxy flavone | 3.78 | y = 6343x + 71.913 | 0.9969 | 0.5-20 | 0.35 | 0.80 | 95.33 |
| Quercetin | 3.05 | y = 68130x - 133639 | 0.9983 | 0.5-20 | 0.39 | 1.19 | 94.31 |
| 3',4,5,7-tetreahydroxy flavone | 2.96 | y = 82255x - 289662 | 0.9985 | 0.5-20 | 0.42 | 1.27 | 86.73 |
| Ligstorside | 2.92 | y = 17918x – 2974.1 | 0.9996 | 0.5-20 | 0.31 | 0.93 | 86.43 |
| Oleocanthal | 2.94 | y = 2384.5x – 845.48 | 0.9980 | 0.5-20 | 0.10 | 0.71 | 85.44 |
| Oleuoside | 2.85 | y = 4579x – 3169.1 | 0.9973 | 0.5-20 | 0.15 | 0.45 | 92.87 |
| Oleuropein | 2.69 | y = 14833x - 5282.3 | 0.9993 | 0.5-20 | 0.14 | 0.35 | 96.88 |
| Oleacein | 2.54 | y = 2641.1x – 700.45 | 0.9997 | 0.5-20 | 1.56 | 2.16 | 93.30 |
| Salicylic acid | 2.44 | y = 12405x + 9607 | 1.000 | 0.5-20 | 0.21 | 1.07 | 85.18 |
| Apigenin 7-glucoside | 2.41 | y = 26819x - 23144 | 0.9977 | 0.5-20 | 0.52 | 1.69 | 94.92 |
| Luteolin 7-glucoside | 2.34 | y = 20573x – 7756.4 | 0.9989 | 0.5-20 | 0.52 | 1.57 | 90.33 |
| Ellagic acid | 2.22 | y = 105392x - 18766 | 0.9998 | 0.5-20 | 0.17 | 0.52 | 90.50 |
| 2-hydroxy-cinnamic acid (trans) | 2.19 | y = 142725x - 13588 | 0.9996 | 0.5-20 | 0.19 | 0.71 | 93.13 |
| Ferulic acid | 1.91 | y = 29993x - 6501.2 | 0.9997 | 0.5-20 | 0.28 | 0.86 | 86.75 |
| 3-methoxy-4-hydroxy cinnamic acid | 1.78 | y = 65581x - 16482 | 0.9999 | 0.5-20 | 0.17 | 0.51 | 89.78 |
| p-coumaric acid | 1.44 | y = 85755x - 34844 | 0.9996 | 0.5-50 | 0.32 | 0.98 | 89.94 |
| Vanillin | 1.22 | y = 117082x - 469630 | 0.9973 | 0.5-20 | 0.10 | 0.30 | 89.45 |
| 2,4 dihydroxy benzoic acid | 0.82 | y = 28900x - 20637 | 0.9988 | 0.5-20 | 0.48 | 1.15 | 96.75 |
| Vanillic acid | 0.78 | y = 49621x - 52631 | 0.9931 | 0.5-20 | 0.45 | 1.12 | 88.49 |
| Catechin | 0.66 | y = 5883.3x - 15172 | 0.9944 | 0.5-20 | 0.88 | 2.65 | 90.57 |
| 4-hydroxy-benzoic acid | 0.54 | y = 40721x + 91209 | 0.9994 | 0.5-20 | 0.40 | 1.23 | 98.88 |
| 2-(-4- hydroxy phenyl) ethanol | 0.51 | y = 28088x + 75468 | 1.000 | 0.5-100 | 0.37 | 1.13 | 99.99 |
| 3 Hydroxytyrosol | 0.31 | y = 20898x – 4825.5 | 0.9996 | 0.5-200 | 0.26 | 0.78 | 90.16 |
| Gallic Acid | 0.26 | y = 30973x - 13376 | 0.9976 | 0.5-20 | 0.12 | 0.45 | 98.46 |

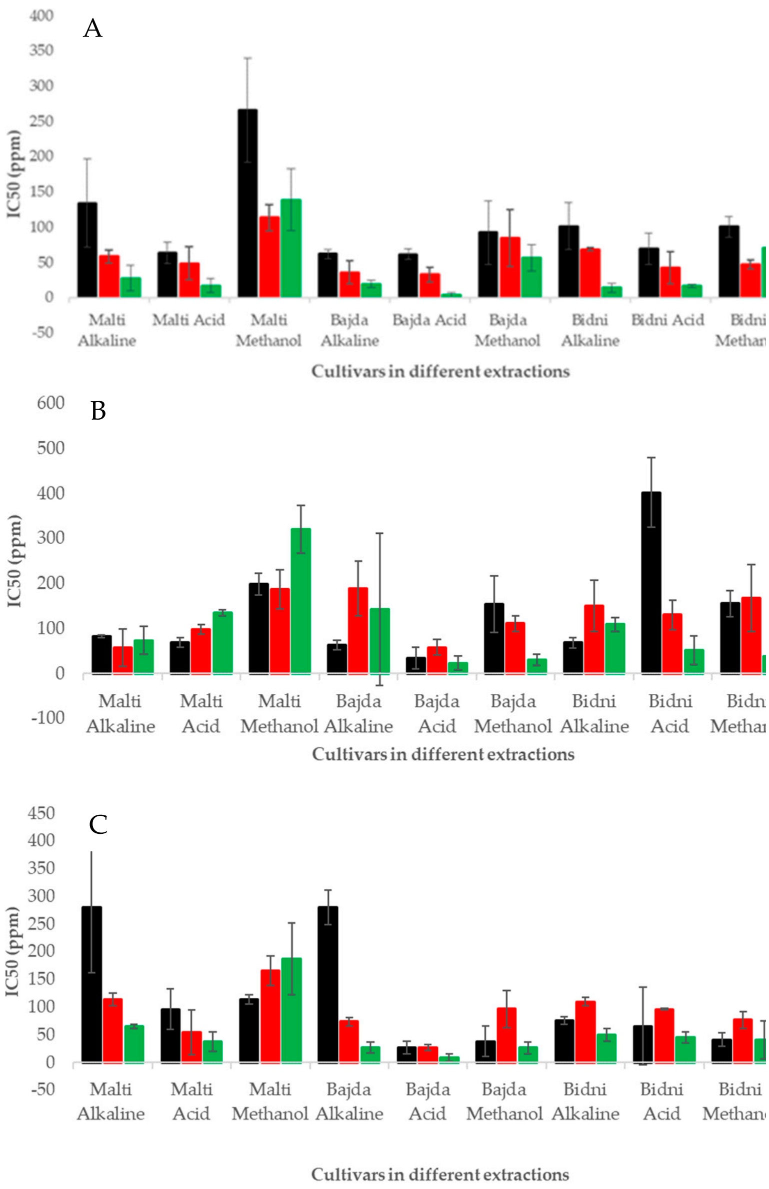


**Figure S4: IC$_{50}$'s of (A) NB4r2, (B) HL-60 and (C) KG-1a cell lines treated with olive mill waste extracts at 24 (black), 48 (red) and 72 (green) h of exposure.**

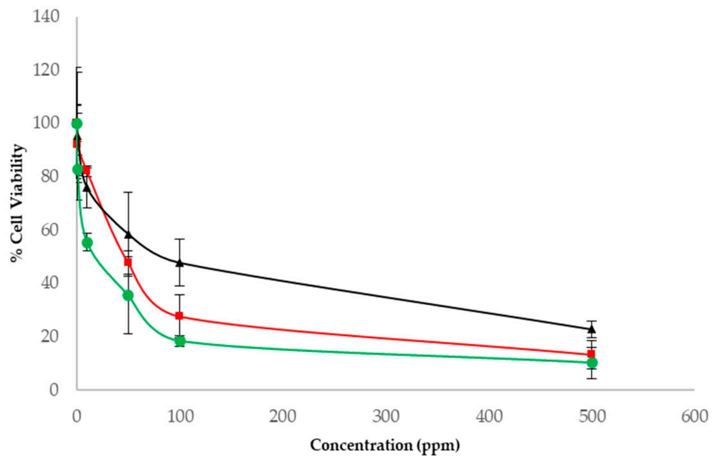


**Figure S5: The percentage cell viability against concentration graph of 'Bajda' cultivar acid (red), methanol (black) and alkaline (green) extraction on NB4r2 cell line at 48 hours of exposure.**

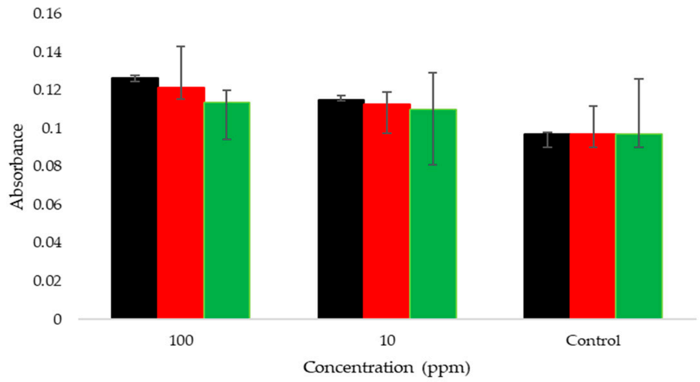


**Figure S6: Caspase-3 activity on KG-1a cell lines after 24 h of exposure using different concentrations (100 and 10 ppm) for 'Bidni' (black), 'Bajda' (red) and 'Malti' (green) extracts with the addition of a control.**

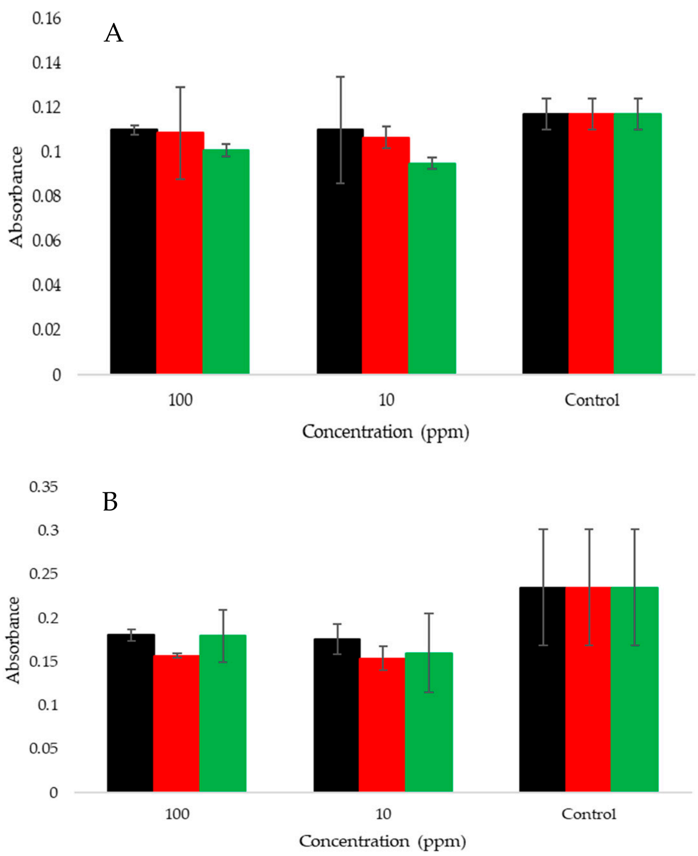


**Figure S7: BCL-2 content on KG-1a cell lines after 24 hours (A) and 48 hours (B) of exposure using different concentrations for 'Bidni' (black), 'Bajda' (red) and 'Malti' (green) extracts with the addition of a control.**

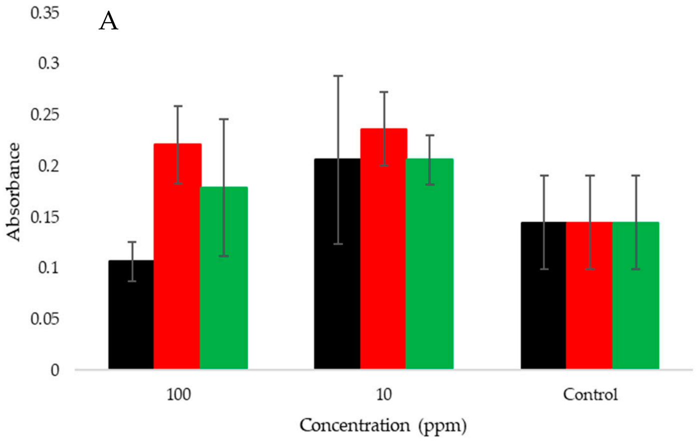


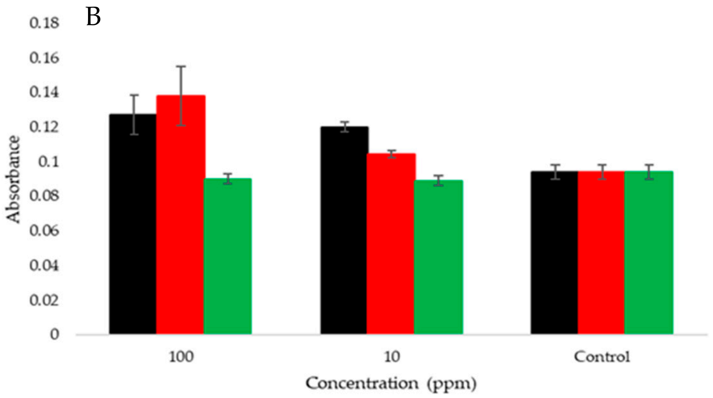


**Figure S8: BAX content on KG-1a cell lines after 24 hours (A) and 48 hours (B) of exposure using different concentrations for 'Bidni' (black), 'Bajda' (red) and 'Malti' (green) extracts with the addition of a control.**

## % Mortality probit

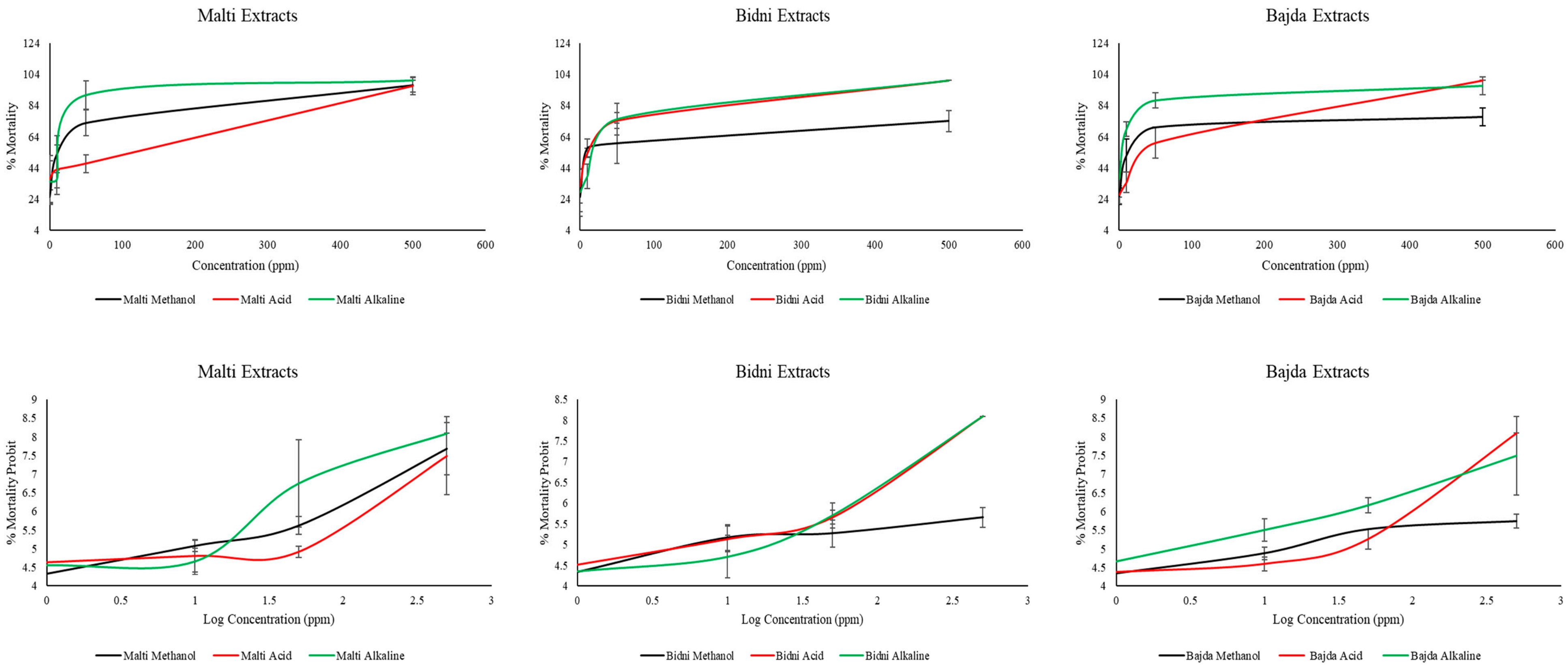


**Figure S9: The percentage mortality probit of olive mill waste extracts at different extraction procedures.**

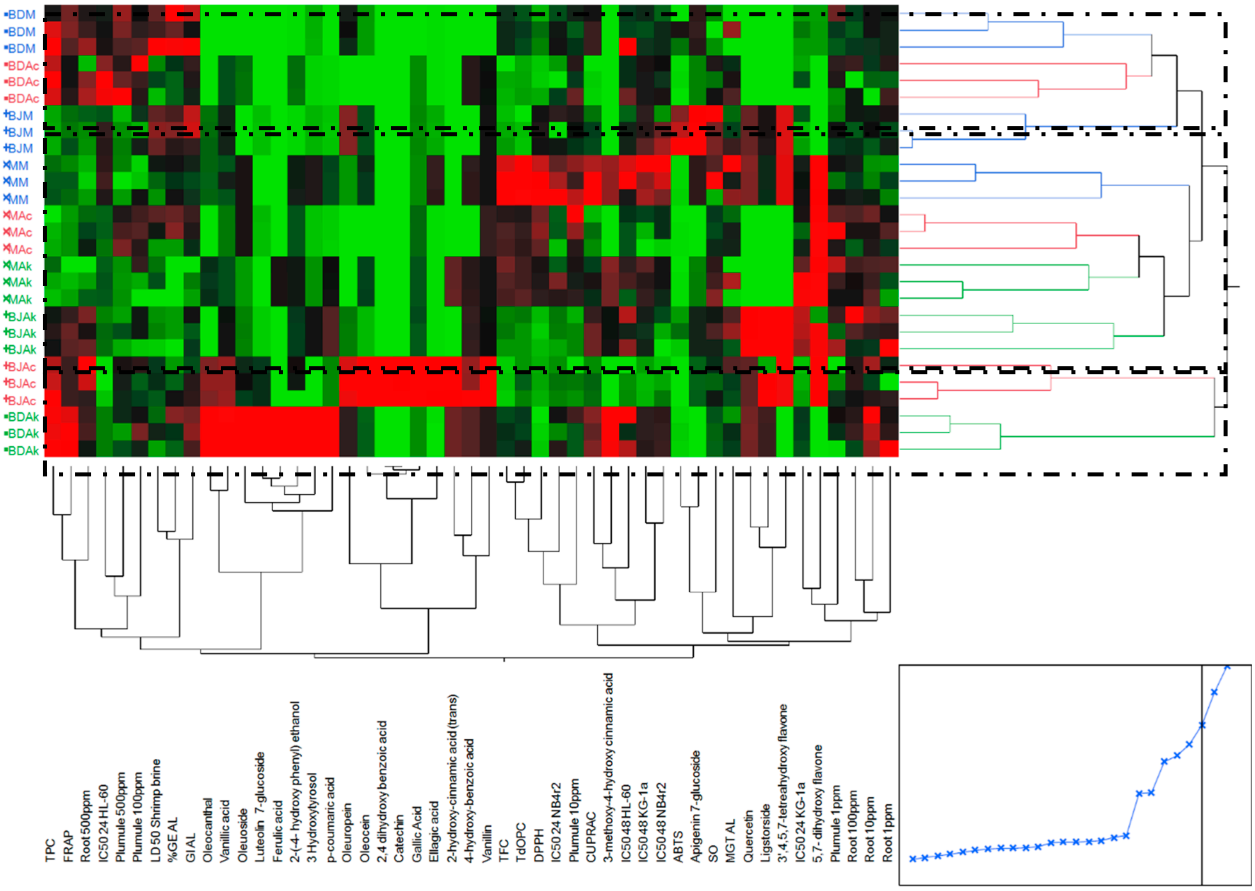


**Figure S10: Two-way cluster analysis, utilizing Ward's method, reveals three distinct clusters with 'Bidni' extracts dominating the first cluster's biochemical profile.**
